# Temporal dynamics of apoptosis-induced proliferation in pupal wing development: implications for regenerative ability

**DOI:** 10.1186/s12915-024-01894-1

**Published:** 2024-04-29

**Authors:** Sara Ahmed-de-Prado, Carlos Estella, Antonio Baonza

**Affiliations:** 1 The Francis Crick Institute, London, NW1 1AT UK; 2 Centro de Biología Molecular Severo Ochoa (CSIC/UAM), C/Nicolás Cabrera 1, Madrid, 28049 Spain

**Keywords:** Apoptosis-induced proliferation, JNK signalling, Regeneration, Imaginal wing discs, Drosophila

## Abstract

**Background:**

The ability of animals to regenerate damaged tissue is a complex process that involves various cellular mechanisms. As animals age, they lose their regenerative abilities, making it essential to understand the underlying mechanisms that limit regenerative ability during aging. *Drosophila melanogaster* wing imaginal discs are epithelial structures that can regenerate after tissue injury. While significant research has focused on investigating regenerative responses during larval stages our comprehension of the regenerative potential of pupal wings and the underlying mechanisms contributing to the decline of regenerative responses remains limited.

**Results:**

Here, we explore the temporal dynamics during pupal development of the proliferative response triggered by the induction of cell death, a typical regenerative response. Our results indicate that the apoptosis-induced proliferative response can continue until 34 h after puparium formation (APF), beyond this point cell death alone is not sufficient to induce a regenerative response. Under normal circumstances, cell proliferation ceases around 24 h APF. Interestingly, the failure of reinitiating the cell cycle beyond this time point is not attributed to an incapacity to activate the JNK pathway. Instead, our results suggest that the function of the ecdysone-responsive transcription factor E93 is involved in limiting the apoptosis-induced proliferative response during pupal development.

**Conclusions:**

Our study shows that apoptosis can prolong the proliferative period of cells in the wing during pupal development as late as 34 h APF, at least 10 h longer than during normal development. After this time point, the regenerative response is diminished, a process mediated in part by the ecdysone-responsive transcription factor E93.

**Supplementary Information:**

The online version contains supplementary material available at 10.1186/s12915-024-01894-1.

## Background

Regeneration is a remarkable ability present across the animal kingdom that enables multicellular organisms to repair damaged tissues and maintain tissue homeostasis [1, 2]. This intricate process involves various cellular mechanisms, including regenerative growth. As animals age, they progressively lose their regenerative abilities, including some salamanders with boundless regenerative capacities. Understanding the underlying mechanisms that limit regenerative ability during aging is a crucial developmental biology question.

During development, intrinsic cellular and physiological changes occur that limit the ability of signaling pathways to promote cellular plasticity and induce regenerative growth, which are crucial to support regeneration [3, 4]. For instance, differentiated cells lose the ability to re-enter the cell cycle, a process necessary for limb regeneration in vertebrates. Tumor suppressor proteins like the Retinoblastoma protein (Rb) likely play a crucial role in maintaining plastic cellular states and cell cycle re-entry. The levels of this factor vary during regeneration in salamanders and across developmental stages in mammals [4]. Nevertheless, experimental evidence in *Xenopus laevis* suggests that the decline in regenerative ability associated with aging, particularly the loss of the capacity to regenerate limb buds, can be delayed or even reversed.

The imaginal wing disc of *Drosophila melanogaster* is a well-established model tissue for studying epithelial damage repair [5–8]. These sac-like structures have the remarkable ability to regenerate during larval stages but lose this ability at the end of the larval stage or during pupal development, which coincides with the cessation of cell proliferation [9–11].

During the larval stage, the cells in the wing disc proliferate asynchronously. However, once the larva-to-pupa transition occurs (0 h after puparium formation (APF), the cells in the pupal wing undergo a G2 phase arrest, which stops their proliferation. By 6 h APF, the majority of pupal wing cells have entered G2 arrest. Between 12–18 h APF, a fraction of these cells resumes the cell cycle and undergo the last round of division before being arrested in G1. By 24 h APF, approximately 95% of the pupal wing cells are arrested in G1 phase and become quiescent before initiating terminal differentiation [12–14].

Steroid hormones play a central role in coordinating the timing of developmental events and significantly affect cell cycle dynamics throughout pupal development. A pulse of the steroid hormone ecdysone during the larval-pupal transition triggers the induction of Broad, which subsequently represses the phosphatase Cdc25 (known as *string* or *stg*), causing a transient G2 arrest at 6 h APF. As ecdysone levels decline, Broad expression decreases, allowing *stg* to be reactivated. This reactivation leads to a rapid G2/M progression during the final round of cell division in pupal wings. This coordinated sequence culminates in a synchronized cell cycle exit around 24 h APF [12, 13] coinciding with a subsequent strong ecdysone pulse.

The exit from the cell cycle at 24 h is triggered by a decrease in the level of Cyclin E (CycE) and a simultaneous increase in the activity of the Rb factor [11]. Rb represses the transcription of genes required for the G1-S transition, including *CycE*, by binding and inhibiting the transcription factor E2F1 [15, 16]. CycE, in turn, promotes the G1-S transition by phosphorylating and inhibiting Rb. Although cell cycle exit occurs around 24 h APF, the positive feedback loop between CycE and E2F1 is maintained until around 36 h APF. Therefore, over-expression of either CycE or E2F1 before this time APF is sufficient to induce proliferation. However, after 36 h, co-expression of *CycE* and *E2F1* is necessary to induce proliferation [11]. The positive feedback between CycE and E2F1 ends around the onset of epigenetic shutdown of regulatory regions of key genes involved in cell cycle control, such as *CycE*, *E2F1*, and *stg*. In contrast to the majority of cell cycle genes, *CycE*, *stg* and *E2F1* are regulated by extensive, intricate regulatory elements that are located upstream of their transcription start site (TSS) or within long introns [17]. Notably, the accessibility of these regulatory elements undergoes a temporal dynamic during metamorphosis. This coincides with changes in the cell cycle. Initially, the accessibility is low during the G2 arrest at 6 h, and subsequently increases at 18 h and 24 h, before closing at 36 h [17]. These changes provide a molecular mechanism for the robust G0 state observed after 36 h of APF. Although the specific chromatin remodelers responsible for the closure of distal regulatory elements at *CycE*, *stg* and *E2F1* during pupal development have not been identified, conserved ecdysone receptor (EcR) binding sites have been identified in these regions [14, 17].

Ecdysone-responsive transcription factors, in particular E93, have been reported to play a pivotal role in orchestrating the temporal changes in chromatin accessibility during the stages of wing development [14, 18]. These factors act as key regulators, presumably influencing the dynamic opening and closing of chromatin regions in response to ecdysone signalling cues [18].

The c-Jun N-terminal kinase (JNK) signalling pathway has emerged as a critical signal in the process of regeneration. Upon injury, the JNK signalling pathway is initiated at the wound site [19]. This pathway plays a key role in regulating several biological processes associated with regeneration [19–23]. In studies of disc regeneration, inhibition of JNK function was found to negatively affect wound healing and reduce regenerative proliferation [19, 24, 25]. During regeneration, JNK signalling promotes the activation of several downstream pathways, including JAK/STAT and Wingless (Wg) [9, 26–28].

Despite extensive research on regenerative responses during larval stages, our understanding of the regenerative abilities of pupal wings and the mechanisms involved in the decline of regenerative responses remains limited. It is unclear whether the signals activated by tissue damage during larval stages also operate during pupal development.

In this study, we investigated the proliferative response triggered by the induction of cell death during pupal development. Our findings indicate that apoptosis-induced proliferation (AiP) response can be triggered up to 34 h APF. As cell proliferation normally ceases at 20–24 h APF during normal development, these results suggest that cell death can extend the proliferative phase of cells in the wing during pupal development. We found that after 34 h APF cell death is not sufficient to induce proliferation. Our data suggest that the inability to reactivate the cell cycle after this time point is not due to a failure in the activation of the JNK pathway. Rather, the activity of the ecdysone-responsive transcription factor E93 appears to be required to limit the proliferative response during pupal development.

## Results

### Ionizing Irradiation (IR) can trigger an apoptosis-induced proliferation response up to 30–35 h APF

**Fig. 1:**
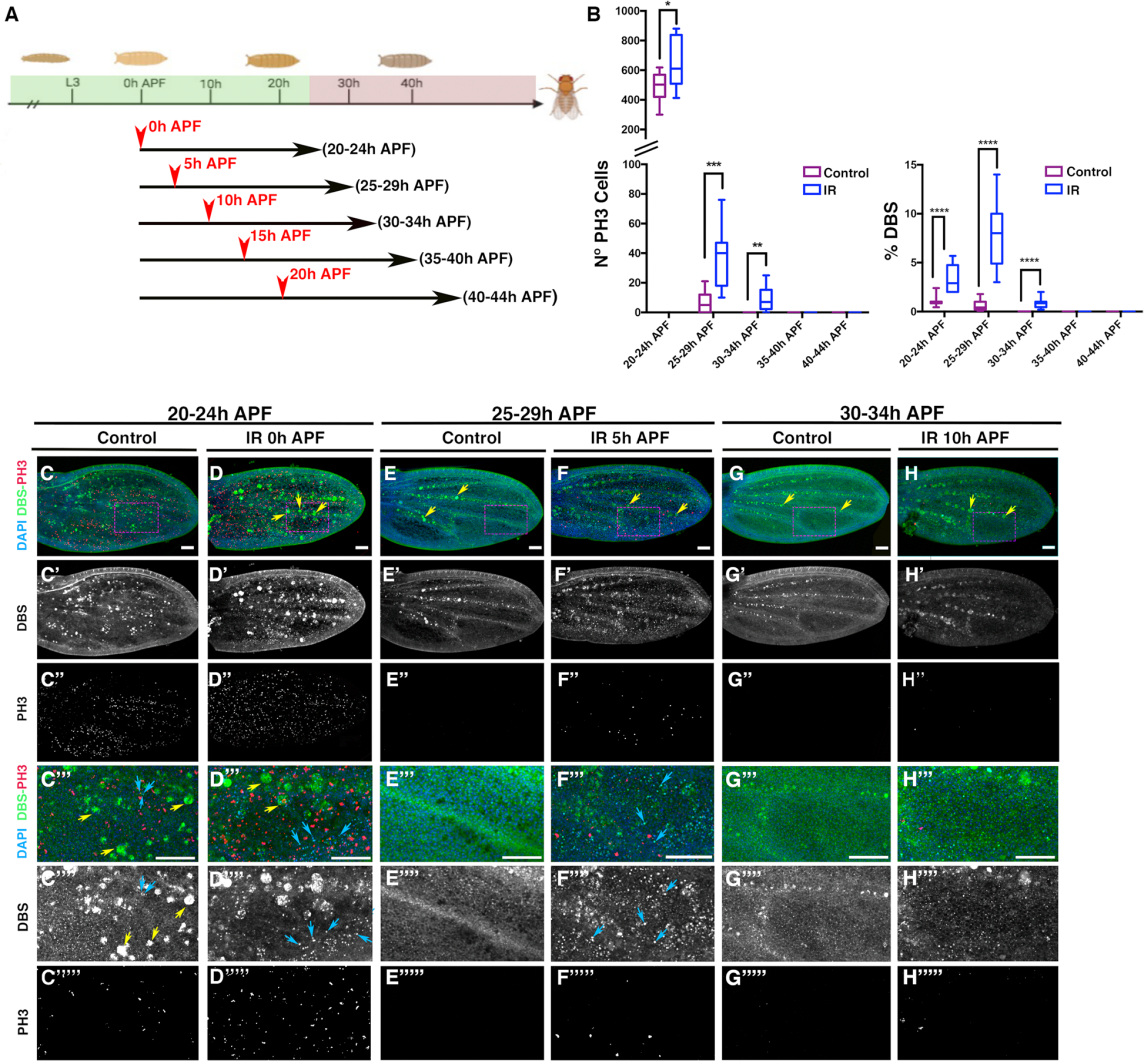
Proliferative and apoptotic response in the pupal wing induced by X-ray irradiation at different times after puparium formation. **A** Schematic diagram of the exposure times used in this experiment. The proliferative period of the pupal wing is indicated by the green color, while the period after cell cycle exit is indicated by the red color. Selected 0–4 h old pupae were irradiated for 8 min at 0 h APF (IR 0 h APF), 5 h later (IR 5 h APF hours), at 10 h APF (IR 10 h APF), at 15 h APF (IR 15 h APF) and at 20 h APF (IR 20 h APF). Pupal wings were analyzed 20 h after exposure. **B** One graph shows the number of mitotic cells (PH3-positive) in both control and irradiated pupal wings at different times APF, and the other the percentage of the region between vein 5 and the posterior wing margin that shows *DBS histone-GFP* nuclear staining (see Methods). (C–C’’’’’, E-E’’’’’ and G-G’’’’’) *P{Tub-DBS-S-H2Av-GFP* control pupal wing at 20–24 h (C–C’’’’’), 25–29 h (E-E’’’’’) and 30–34 h (G-G’’’’’) APF. (D-D’’’’’, F-F’’’’’ and H–H’’’’’) *P{Tub-DBS-S-H2Av-GFP* Pupal wing irradiated at 0 h APF and analyzed at 20–24 h (D-D’’’’’), irradiated at 5 h APF and analyzed at 25–29 h (F-F’’’’’) and irradiated at 10 h APF and analyzed at 30–34 h APF (H–H’’’’’). Note that while there are few or no dividing cells in the control pupal wings at 25-29 h and 30-34 h APF respectively, cell proliferation is sustained in the irradiated pupal wings at the same time point compare E-E’’’’’and G-G’’’’’ with, F-F’’’’’ and H–H’’’’’. Pupal wings were stained with anti-PH3 antibody and DAPI. *DBS-Histone-GFP* nuclear staining indicates the presence of apoptotic cells, comparing the nuclear expression of *DBS-Histone-GFP* (blue arrows in C’’’, C’’’’, D’’’, D’’’’, F’’’ and F’’’’ with the expression of this reporter in presumptive hemocytes (yellow arrows in C’’’, C’’’’, D, D’’’, E, F, G and H). In non-apoptotic cells, *DBS-GFP* is expressed in the membranes of the cells. Images C’’’-H’’’’’ correspond to high magnification of the region indicated by a magenta rectangle in images **C**-**H**. Statistical significance was determined using multiple comparation t-Test student (Mann–Whitney test) **** *p* < 0.0001. ***p < 0.001, **p < 0.01 *p < 0.05. Control 20–24 h *n* = 12, 25–29 h *n* = 11, 30–34 h *n* = 11, 35–39 h *n* = 15 and 40–44 h *n* = 15. Irradiated 20–24 h *n* = 13, 25–29 h *n* = 13, 30–34 h *n* = 12, 35–39 h *n* = 11 and 40–44 h *n* = 11. Error bars represent 99% percentile. White scale bar, 50 μm

We analyzed the proliferative and apoptotic response of pupal wing cells following X-ray irradiation during pupal development. Pupae were collected at 4-h intervals (see Methods) and allowed to mature to different developmental stages. They were then irradiated at five specific times: immediately after pupation 0 h (IR 0 h APF), 5 h later (IR 5 h APF hours), at 10 h (IR 10 h APF), at 15 h (IR 15 h APF) and at 20 h APF (IR 20 h APF) (Fig. 1A). Analysis of irradiated pupae was performed 20 h after irradiation. Given the 4-h intervals of pupal collection, the analysis periods were defined as 20–24 h (Fig. 1C-D’’’’), 25–29 h (Fig. 1E–F’’’’’), 30–34 h (Fig. 1G-H’’’’’), 35–39 h (Additional file 1: Fig. S1 A-B’’’’) and 40-44 h (Additional file 1: Fig. S1 C-D’’’’’). The developmental temperature was kept constant at 25˚C.The mitotic marker phospho-histone 3 (PH3) was used to assess the proliferative response. To visualize apoptotic cells, we used the Drice-based sensor (DBS) (CD8-DriceC211A-shortHistone-GFP; hereafter DBS). This reporter provides an early indication of cell death in *Drosophila*. In the absence of apoptotic signals, this construct remains tethered to cell membranes outside the nucleus. However, when the apoptotic pathway is activated, Histone-GFP translocates into the nucleus, indicating the presence of apoptotic cells [29]. At 20-24 h APF, both irradiated and non-irradiated pupal wings showed a high number of mitotic and apoptotic cells (Fig. 1B and C-D’’’’’). However, irradiated pupal wings showed a significant increase in both mitotic and apoptotic cells compared to non-irradiated samples (Fig. 1B). At 25-29 h, non-irradiated pupal wings contained sporadic mitotic cells and the presence of apoptotic cells was reduced compared to the earlier stage (Fig. 1B and E-E’’’’’). However, irradiated pupal wings still showed a substantial presence of apoptotic cells, as indicated by Histone-GFP in numerous nuclei (blue arrows in Fig. 1F’’’ and F’’’’), together with a relatively high number of mitotic cells (Fig. 1B and F-F’’). At 30-34 h, there are no mitotic cells in the control non-irradiated pupal wing, but we still find few dividing cells in the irradiated pupal wings, and the percentage of apoptotic cells in these pupal wings is still elevated compared to the control of non-irradiated wings (Fig. 1B and G-H’’’’’). Pupal wings examined at 35–39 h and 40–44 h APF revealed the absence of mitotic cells or significant apoptotic cells in both control and irradiated pupal wings (Fig. 1B and Additional file 1: Fig. S1 A-D’’’’). These results suggest that irradiation-induced apoptosis can prolong the proliferative period up to 30–34 h APF. They also suggest that wing cells became insensitive to radiation-induced apoptosis during pupal development.

**Fig. 2:**
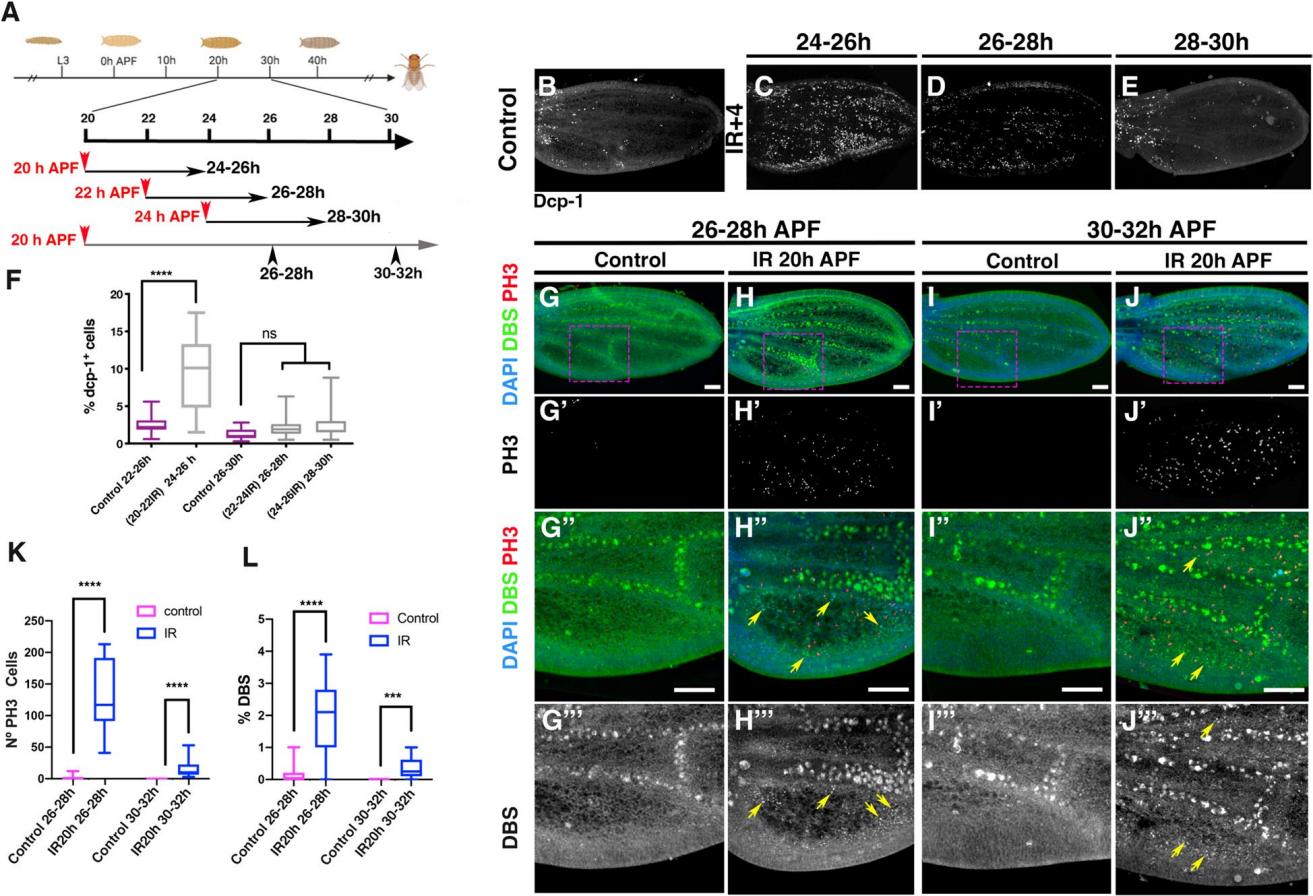
Cell death induction by irradiation during pupal development. **A** A schematic diagram illustrates the exposure times used in this experiment. Pupae selected at 0–2 h interval (see Methods) were allowed to progress to 20 h, 22 h and 24 h APF before being exposed to 8 min of irradiation. Analysis was performed 4 h after irradiation. Given the 2 h interval chosen, the age of the pupae at the time of analysis correspond to 24–26 h, 26–28 h and 28–30 h APF. In another set of experiments (grey line), *P(Tub-DBS-S-H2Av-GFP)* pupae were irradiated at 20 h APF and analyzed 6 h later at 26–28 h or 10 h later at (30–32 h) APF. **B** Control pupal wing at 24–26 h APF. **C**, **D** and **E** Pupal wing of pupae exposed at 20 h APF and analyzed at 24-26 h (**C**), exposed at 22 h APF and analyzed at 26-28 h (**D**) and exposed at 24 h APF and analyzed at 28-30 h (**E**) APF. Pupal wings were stained with anti-Dcp-1 antibody. **F** The graph shows the percentage of apoptotic cells (Dcp-1 positive) in control and irradiated pupae at different times APF. Error bars represent 99% percentile. Statistical analysis was conducted using One-way ANOVA Tukey's test **** *p* < 0.0001. Control: 22–26 h *n* = 25, and 26–30 h *n* = 15. Irradiated: 24–26 h *n* = 11, 26–28 h *n* = 11 and 28–30 h *n* = 7. (G-G’’’ and I-I’’’) *P{Tub-DBS-S-H2Av-GFP* control pupal wing at 26–28 h (G-G’’’) and 30–32 h (I-I’’’) APF. (H–H’’’ and J-J’’’) *P(Tub-DBS-S-H2Av-GFP)* pupal wing exposed at 20 h APF and analyzed at 26-28 h (H–H’’’) and 30-22 h (J-J’’’). Pupal wings were stained with anti-PH3 antibody and DAPI. *DBS-Histone-GFP* nuclear staining indicates the presence of apoptotic cells (yellow arrows in H’’-H’’’ and J’’-J’’’). Images G’’-J’’’ correspond to high magnification of the region indicated by a magenta rectangle in images **G**-**J**. **K** The graph shows the number of mitotic cells (PH3-positive) in both control and irradiated pupae at different times APF. **L** The graph shows the percentage of the region between vein 5 and the posterior wing margin that shows *DBS histone-GFP* nuclear staining. Statistical analysis was performed using the student t-test for multiple comparisons. Statistical significance was determined using multiple comparation t-Test student (Mann–Whitney test) **** *p* < 0.0001. ***p < 0.001, **p < 0.01 *p < 0.05. **K** Control 26–28 h *n* = 15, IR 20 h 26–28 h *n* = 12, Control 30–32 h *n* = 15, IR20h 30–32 h *n* = 12. **L** Control 26–28 h *n* = 15, IR 20 h 26–28 h *n* = 11, Control 30–32 h *n* = 15, IR20h 30–32 h *n* = 12

In order to precisely determine the window during which pupal wing cells become unresponsive to irradiation, we selected pupae aged 0–2 h (see Methods). We then allowed these pupae to progress to 20 h, 22 h and 24 h APF before exposing them to irradiation at these defined time points. The age of the pupae corresponded to 20–22 h, 22–24 h and 24–26 h APF due to the choice of a 2-h interval. The apoptotic response was analysed 4 h after exposure. Our results showed a significantly higher number of apoptotic cells in wings of pupae irradiated at 20 h and examined 4 h later compared to control wings (Fig. 2B-C and F). We did not observe an increase in the number of apoptotic cells in pupal wings of animals irradiated at 22–24 h and 24–26 h APF (Fig. 2D-E and F). Therefore, these results suggest that pupal wing cells acquire radio-resistance around 22–24 h APF. Intriguingly, our observations indicate that while irradiation at 20 h APF can induce apoptosis, it does not simultaneously increase proliferation in the pupal wing analyzed 20 h later (Fig. 1 and Additional file 1: Fig. S1). This discrepancy could either be due to the fact that the proliferative response is never initiated despite the induction of apoptosis at this time, or that the response is initiated but not sustained, halting around 34 h APF, the observed time point when cell proliferation ceases. To investigate this further, we examined the proliferation pattern alongside the apoptotic response at different times in pupae exposed to irradiation at 20 h APF. For this purpose, we used the DBS construct as an early reporter of apoptosis. We analyzed the apoptotic and proliferative responses at 6 h after irradiation, a time when mitotic arrest is known to be released after at least 3 h of cell arrest induced by irradiation [30, 31]. Our results showed a significant increase in apoptotic cells (Fig. 2H–H’’’ and L, yellow arrows indicate apoptotic cells) in irradiated pupae compared to control wings (Fig. 2G-G’’’). Interestingly, we also observed a robust proliferative response in these irradiated wings, characterized by a significant increase in the number of mitotic cells (Fig. 2H–H’’ and K).

Further analysis 10 h after irradiation showed that proliferation was significantly higher than in control wings, although lower than at the previous time point (Fig. 2I-J’’’ and K). Notably, we consistently observed an increased level of apoptosis compared to controls (Fig. 2J’’-J’’’ yellow arrows and L). These results strongly suggest that irradiation of pupal wings between 20–22 h induces apoptosis and initiates a proliferative response that persists until 30–34 h APF. Our results also suggest that pupal wing cells acquire radioresistance around 22–24 h APF, which coincides with cell exit from the cell cycle during pupal development. It is plausible that cells acquire radioresistance as a result of their non-dividing state. Previous reports suggest that cells that exit the cell cycle or undergo cell cycle arrest are radioresistant [30, 31].

### Genetic ablation expands the period of damage-induced proliferative response during pupal development

Our results indicate that the cells in the pupal wing exhibit insensitivity to IR-induced apoptosis after 24 h (APF). Consequently, it is plausible to consider that the absence of observed proliferative responses in irradiated pupae during the later stages of pupal development may be attributed to the ineffectiveness of IR in inducing apoptosis. To test this, we employed an alternative approach to effectively induce apoptosis. To this end we used the Gal4/UAS binary system with Gal80^ts^ to induce genetic ablation at different times APF. We overexpressed the pro-apoptotic gene *reaper* (*rpr*) in the posterior compartment of the pupal wing using the *hedgehog* (*hh*)*-Gal4* line.

**Fig. 3:**
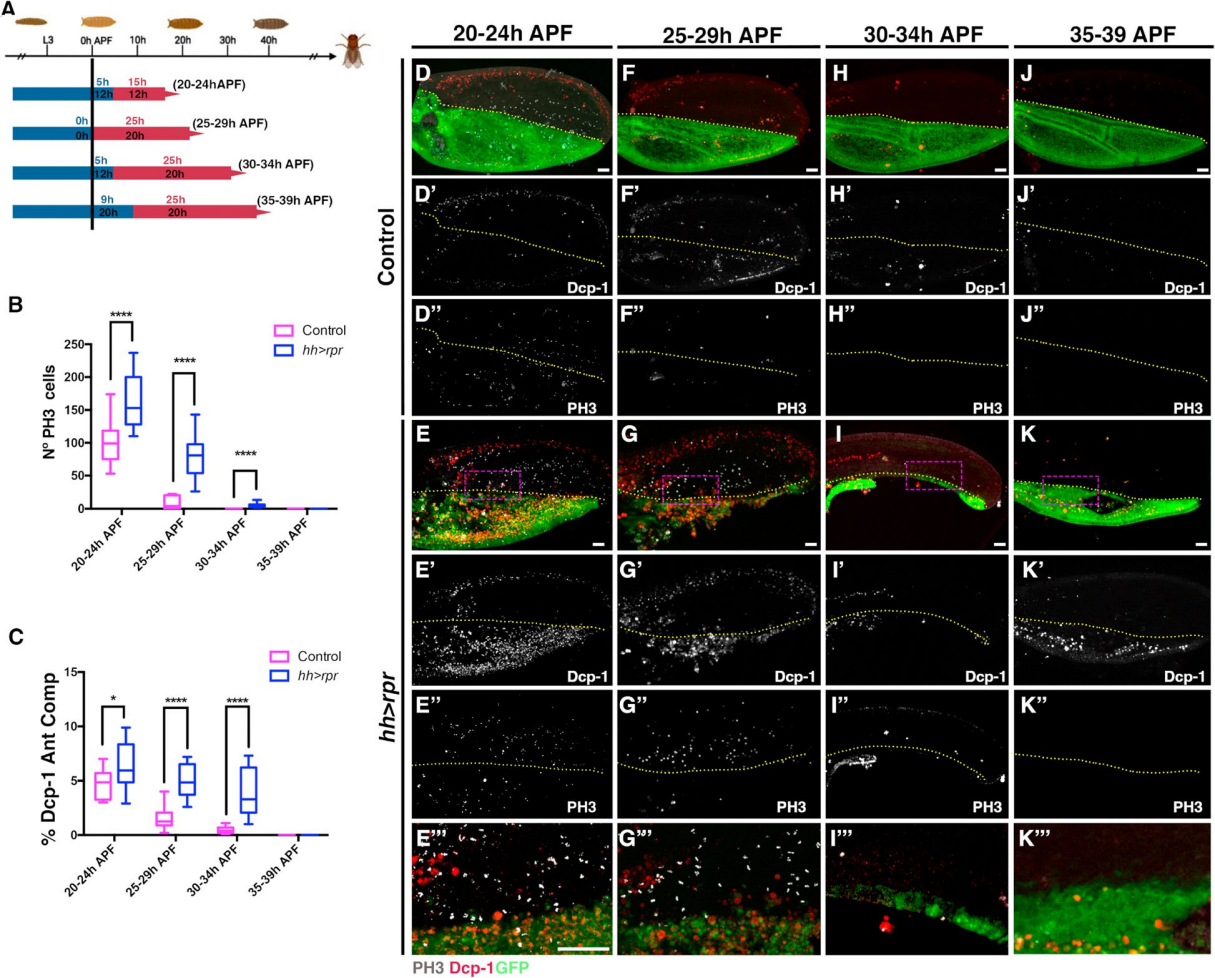
Genetic ablation prolongs the proliferative period of pupal wing. **A** Schematic diagram illustrating the temperature shifts employed in this experiment. *UAS-rpr; hh-Gal4 tub-Gal80*^*ts*^*/* + pupae or control *hh-Gal4 tub-Gal80*^*ts*^*/* + pupae aged 0–4 h were initially kept at 17 °C for varying durations before being transferred to 29 °C to activate *rpr* expression. The blue bar indicates the duration of incubation at 17 °C (black text), while the text shown in blue above indicates the equivalent time at 25 °C. The red bar represents the time incubated at 29 °C, normally 20 h, except for the 20–24 h interval. The equivalence of this time at 25 °C is shown in red above. Developmental stages corresponding to 20–24 h, 25–29 h, 30–34 h, and 35–39 h APF at 25 °C were established following the subsequent temperature shifts: 20–24 h = (12 h at 17 °C + 12 h at 29 °C); 25–29 h = (20 h at 29 °C); 30–34 h = (12 h at 17 °C + 20 h at 29 °C); and 35–39 h = (20 h at 17 °C + 20 h at 29 °C). **B** The graph shows the number of mitotic cells (PH3 positive) in both control and damaged pupae at different times APF. **C** The graph shows the percentage of apoptotic cells (Dcp-1 positive) in the anterior compartment of control and damaged pupae at different time intervals. (D-D’’, F-F’’, H–H’’ and J-J’’) Control *hh-Gal4 tub-Gal80*^*ts*^*/* + pupae pupal wing at 20–24 h (12 h at 17 °C + 12 h at 29 °C) (D-D’’), 25–29 h (20 h at 29 °C) (F-F’’), 30–34 h (12 h at 17 °C + 20 h at 29 °C) (H–H’’) and 35–39 h APF (20 h at 17 °C + 20 h at 29 °C) (J-J’’). (E-E’’’, G-G’’’, I-I’’’ and K-K’’’) *UAS-rpr; hh-Gal4; tub-Gal80*^*ts*^ pupae after 20 h of *rpr* overexpression (except for the 20–24 h interval that were 12 h) were examined at developmental stages corresponding to the following time intervals at 25 °C, 20–24 h (12 h at 17 °C + 12 h at 29 °C) (E-E’’’), 25–29 h (20 h at 29 °C) (G-G’’’), 30–34 h (12 h at 17 °C + 20 h at 29 °C) (I-I’’’) and 35–39 h APF (20 h at 17 °C + 20 h at 29 °C) (K-K'''). Pupal wings were stained with anti-PH3 (grey) and anti-Dcp-1 (red). GFP is shown in green. Images E’’’, G’’’,I’’’ and K’’’ correspond to high magnification of the region indicated by a magenta rectangle in images **E**, **G**, **I** and **K**. Yellow dotted lines indicate the boundary between the anterior and posterior compartments. Statistical analysis was conducted using multiple comparation t-Test student (Mann–Whitney test) **** *p* < 0.0001. **B** Control: 20–24 h *n* = 13, 25–29 h *n* = 15. 30–34 h *n* = 15 and 35–39 h *n* = 15. Ablated *UAS-rpr; hh-Gal4; tub-Gal80*^*ts*^: 20–24 h *n* = 15, 25–29 h *n* = 13. 30-34 h *n* = 15 and 35–39 h *n* = 11. **C** Control: 20–24 h *n* = 12, 25–29 h *n* = 12, 30–34 h *n* = 11 and 35-39 h *n* = 11. Ablated *UAS-rpr; hh-Gal4; tub-Gal80*^*ts*^: 20–24 h *n* = 10, 25–29 h *n* = 6, 30–34 h *n* = 10 and 35–39 h *n* = 14. White scale bar 50 μm

To ensure accurate and reliable results, we modified our protocols to account for differences in Drosophila development at different temperatures (see Methods, Fig. 3A). We selected pupae at 0–4 h APF (see Methods), reared them at 17ºC for varying lengths of time, and then transferred them to 29ºC to activate *rpr* expression for 20 h (except for the 20–24 h interval, see below and Fig. 3A). Given the different temperature conditions used in our experiments and the observed discrepancies in developmental rates at these different temperatures (development is 2.5 times slower at 17 °C and 1.3 times faster at 29 °C, see Methods), we adjusted the duration of pupal incubation at 17 °C to match the developmental stages observed at 25 °C to ensure consistency. To assess the rate of development at different temperatures (see Methods), we considered only two time points: the onset of pupariation and eclosion. It should be noted that we assumed that the temperature shift during pupal development would have a uniform effect on developmental rates. Therefore, our correction to define developmental stages equivalent to pupal growth at 25 °C may not be entirely accurate. Given these considerations and the differences in developmental rates observed at different temperatures (see Methods), we assumed that developmental stages equivalent to 20–24 h, 25–29 h, 30–34 h, and 35–39 h APF at 25 °C would be generated following the subsequent temperature shift: 12 h at 17 °C + 12 h at 29 °C would correspond to 20–24 h, 20 h at 29 °C would correspond to 25–29 h at 25 °C, 12 h at 17 °C + 20 h at 29 °C would correspond to 30–34 h at 25 °C, and 20 h at 17 °C + 20 h at 29 °C would correspond to 35–39 h at 25 °C (Fig. 3A). As mentioned above, we overexpressed *rpr* for 20 h in all the intervals analyzed, except for the 20–24 h interval (12 h at 17 °C + 12 h at 29 °C). This adjustment was necessary because a 20-h period at 29 °C would result in a developmental stage at 25 °C exceeding 24 h, according to our corrections. To account for this, we minimized the duration of exposure to 29 °C during this interval. To assess the proliferative and apoptotic response, we then quantified the number of mitotic and dead cells in pupal wings at these time intervals immediately after finishing the temperature shift to 29 °C (see Fig. 3A and Methods).

The transient overexpression of *rpr* controlled by *hh* > *Gal4*, induced a substantial increase in apoptotic cells at all time intervals examined during pupal development, as indicated by the apoptotic marker Dcp-1. This resulted in the partial or total elimination of the posterior compartment (Fig. 3). Interestingly, our observations show that at stages equivalent to 20-24 h (12 h at 17 °C + 12 h at 29 °C), 25-29 h (20 h at 29 °C) and 30-34 h (12 h at 17 °C + 20 h at 29 °C) APF at 25 °C, apoptosis not only increased within the posterior compartment, but also spread non-autonomously into the anterior compartment (Fig. 3C).

At developmental stages equivalents to 20-24 h (12 h at 17 °C + 12 h at 29 °C), 25-29 h (20 h at 29 °C) and 30-34 h (12 h at 17 °C + 20 h at 29 °C) APF at 25 °C, the damaged pupal wings showed a significant increase in cell proliferation compared to the control pupal wings (Fig. 3B and compared D-D’’, F-F’’, H–H’’ with E-E’’, G-G’’ and I-I’’). At 25–29 h (20 h at 29 °C), a few mitotic cells were detected in the control wings, whereas a significantly higher number of mitotic cells remained in the damaged wings (Fig. 3B and F-G’’’). Similarly, at 30–34 h APF (12 h at 17 °C + 20 h at 29 °C), mitotic cells were consistently present in the damaged wings but absent in the control wings (Fig. 3B and H-I’’’). After 35–39 h APF (20 h at 17 °C + 20 h at 29 °C), no dividing cells were observed in either control or damaged wings (Fig. 3B and J-K’’’).

Overexpression of *rpr* consistently induces apoptosis at all time intervals analysed. Therefore, the absence of a proliferative response after 35 h APF cannot be attributed solely to the absence of apoptosis. Our results suggest a sequence in which apoptosis initiates a proliferative response that lasts up to 30–34 h APF (12 h at 17 °C + 20 h at 29 °C). This response shows significant strength up to 30 h, then gradually diminishes and finally ceases by 34 h APF. Together, our data suggest that mitotic signals generated in the damaged area potentially prolong pupal wing cell proliferation, but this extension is only effective until 30–34 h APF (12 h at 17 °C + 20 h at 29 °C).

In summary, our study provides evidence that the duration of the proliferative response in pupal wing cells is limited and can be influenced by proliferative signals produced by dead cells.

### JNK signalling is activated in response to damage in late stages of pupal development

The JNK pathway is a key player in initiating apoptosis-induced proliferative responses during larval stages and serves as a critical regulator of regenerative growth [19, 24, 25]. Its activity is evident at the leading edge of healing tissues shortly after genetic or surgical ablation and persists and expands during and after wound healing [5, 9, 10, 19].

Upon JNK activation, there is a localized increase in Wingless (Wg, the Drosophila homolog of Wnt1) and Decapentaplegic (Dpp, a Drosophila homolog of BMPs) [32–36]. Damage-induced activation of *wg* relies on the *DRMSWNT* enhancer, which stimulates the expression of both *wg* and *wnt6* to facilitate regeneration [36]. JNK signalling directly regulates the activity of this enhancer in response to damage during larval stages [36, 37]. Interestingly, its activity decreases at the end of the larval period [36].

We investigated the evolution of the JNK pathway and *DRMSWNT* enhancer activity in response to the induction of cell death after metamorphosis. To this end, we analyzed the expression of *TRE-RFP* [38] and *DRWNT-GFP* reporters [36] following overexpression of *rpr* under the control of *hh-Gal4* at various times APF. The *DRWNT-GFP* reporter derived from a fragment of the *DRMSWNT* enhancer and shows robust damage-responsive expression during larval development and maintains its activity throughout the larval stage [36].

We selected pupae within the 0–4 h APF range (Methods) and reared them at different times at 17 °C before transferring them to 29 °C to induce *rpr* expression for 20 h. We followed the same protocol as previously described for the genetic ablation experiment to analyse developmental stages equivalent to 20-24 h (12 h at 17 °C + 12 h at 29 °C), 25-29 h (20 h at 29 °C), 30-34 h (12 h at 17 °C + 20 h at 29 °C), 35–39 h (20 h at 17 °C + 20 h at 29 °C) and 45–49 h (48 h at 17 °C + 20 h at 29 °C) APF at 25 °C (Fig. 3A).

**Fig. 4:**
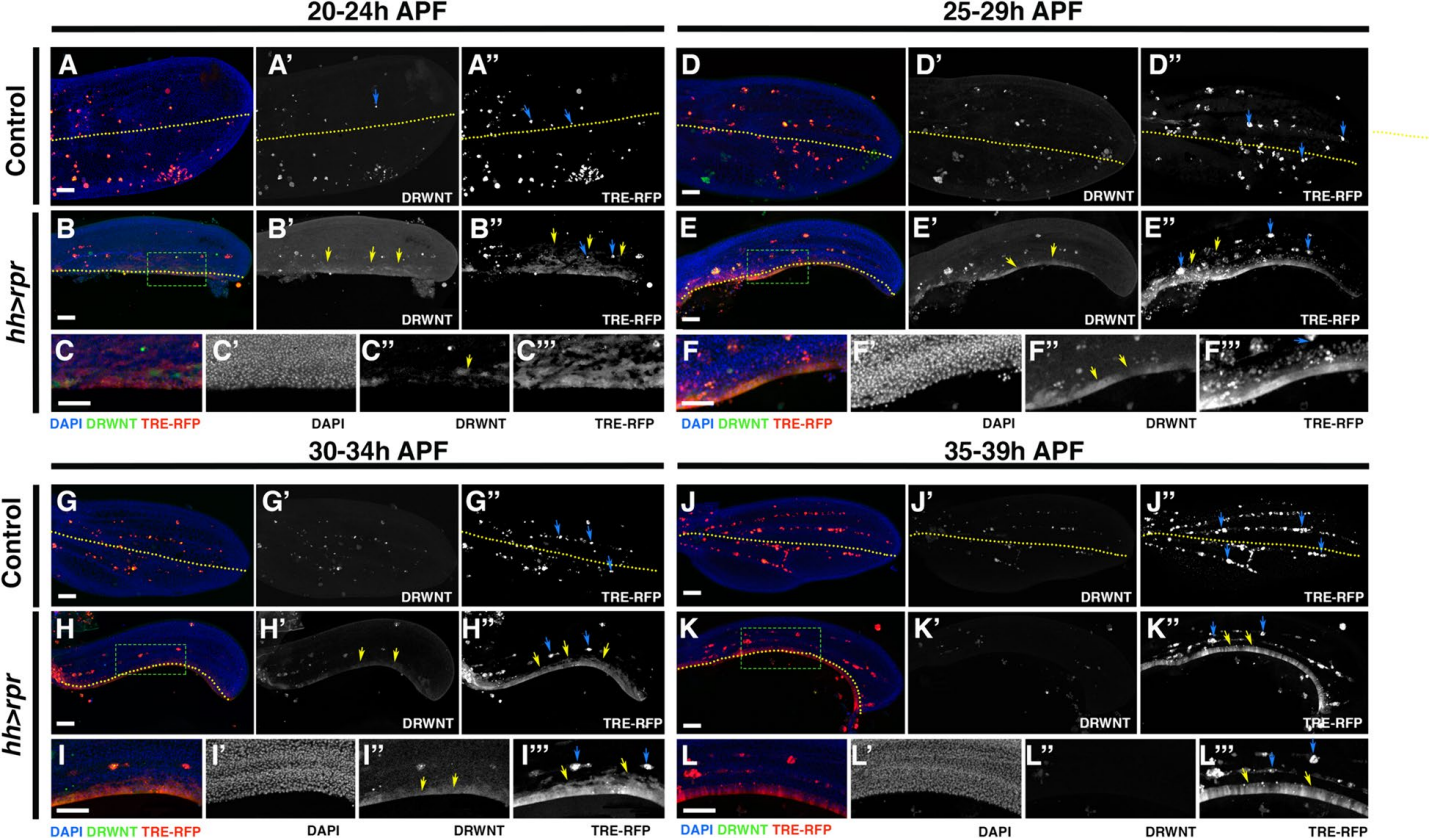
Patterns of JNK and DRWNT activation following genetic ablation of the posterior compartment in the pupal wing. We used the same protocol as described in Fig. 3A to induce the expression of *rpr* in *UAS-rpr; DRWNT-GFP hh-Gal4; tub-Gal80*^*ts*^* TRE-RFP* pupae. (A-A’’, D-D’’, G-G’’ and J-J’’) *DRWNT-GFP hh-Gal; tub-Gal80*^*ts*^* TRE-RFP* control pupal wings examined at developmental stages corresponding to the following time intervals at 25 °C: 20–24 h (12 h at 17 °C + 12 h at 29 °C) (A-A’’), 25–29 h (20 h at 29 °C) (D-D’’), 30–34 h (12 h at 17 °C + 20 h at 29 °C) (G-G’’) and 35–39 h APF (20 h at 17 °C + 20 h at 29 °C) (J-J’’) APF. (B-C’’’, E–F’’’, H-I’’’ and K-L’’’) *UAS-rpr; DRWNT-GFP hh-Gal4; tub-Gal80*^*ts*^* TRE-RFP* pupal wing after 20 h of *rpr* overexpression were examined at developmental stages corresponding to the following time intervals at 25 °C: 20–24 h (12 h at 17 °C + 12 h at 29 °C) (B-C’’’), 25–29 h (20 h at 29 °C) (E–F’’’), 30–34 h (12 h at 17 °C + 20 h at 29 °C) (H-I’’’) and 35–39 h APF (20 h at 17 °C + 20 h at 29 °C) (K-L’’’) APF. Images C–C’’’, F-F’’’, I-I’’’ and L-L’’’ correspond to high magnification of the area marked with a green rectangle in **B**, **E**, **H** and **K**. The expression of *TRE-RFP* is shown in red, *DRWNT-GFP* in green and DAPI in blue. Note that in control pupal wings the expression of the *TRE-RFP* reporter is restricted to presumptive hemocytes (blue arrows in A’’, D’’, G’’ and J’’), however in ablated pupal wings *TRE-RFP* reporter is expressed not only in presumptive hemocytes (blue arrows in B’’, E’’, F’’’, H’’, I’’’, K’’ and L’’’) but also in cells within the wing epithelium, particularly in regions adjacent to the wound, as indicated by DAPI staining (yellow arrows in B’’, E’’, H’’, I’’’, K’’ and L’’’). At 20-24 h (B-C’’’) and 25-29 h (E–F’’’), *TRE-RFP* expression expanded into the anterior compartment, reaching anterior vein 3 (yellow arrows in B’’ and E’’). The *DRWNT-GFP* reporter showed activity in damaged discs at 20-24 h (12 h at 17 °C + 12 h at 29 °C) (yellow arrows in B’ and C’’), at 25-29 h (20 h at 29 °C) (yellow arrows in E’ and F’’) and at 30-34 h (12 h at 17 °C + 20 h at 29 °C) (yellow arrows in H’ and I’’), but not at 35-39 h (20 h at 17 °C + 20 h at 29 °C) (J-L’’). This reporter is also expressed in some presumptive hemocytes in control pupal wing (A’). The yellow dotted line indicates the boundary between the anterior and posterior compartment. White scale bar 50 μm

In all intervals analyzed, we consistently observed *TRE-RFP* expression adjacent to the damaged region, in addition to numerous presumptive hemocytes mainly localized to the wing veins (blue arrows in Fig. 4B’’, E’’, F’’’, H’’, I’’’, K’’ and L’’’). This differed from controls in which the reporter activity remained restricted to presumptive hemocytes in the wing veins (blue arrows in Fig. 4A’’, D’’, G’’ and J’’). Notably, at very late stages (35-39 h APF (20 h at 17 °C + 20 h at 29 °C) and 45-49 h APF (48 h at 17 °C + 20 h at 29 °C), *TRE-RFP* reporter expression persisted even though cell proliferation was no longer activated due to cell death induction (Fig. 4J-L’’’ and Additional File 2: Fig. S2). During 20-24 h (12 h at 17 °C + 12 h at 29 °C), and 25-29 h (20 h at 29 °C) intervals *TRE-RFP* expression expanded into the anterior compartment, reaching anterior vein 3 (yellow arrows in Fig. 4B’’ and E’’). This is consistent with previous findings indicating that in response to damage, expression of this reporter extends to the area adjacent to the damaged region [19]. However, after 30–34 h APF (12 h at 17 °C + 20 h at 29 °C), *TRE-RFP* expression was restricted to the damaged region adjacent to the anterior/posterior boundary of the compartment (yellow arrows in Fig. 4H’’, I’’’, K’’ and L’’’).

The *DRWNT-GFP* reporter showed activity in damaged discs at early stages 20-24 h (12 h at 17 °C + 12 h at 29 °C), 25-29 h (20 h at 29 °C), and 30-34 h (12 h at 17 °C + 20 h at 29 °C) (Fig. 4A-I’’’), but not at 35–39 h (20 h at 17 °C + 20 h at 29 °C) and 45–49 h (48 h at 17 °C + 20 h at 29 °C) APF (Fig. 4J-L’’ and Additional File 2: Fig. S2). Its expression remained consistently restricted to the boundary between the anterior and posterior compartments (yellow arrows in Fig. 4, B', C’’, E’, F’’, H’, and I’’). In uninjured control wings, reporter expression was exclusively observed in some presumptive hemocytes (blue arrows in Fig. 4A-A’, D-D’, G-G’, and J-J’). Interestingly, while previous reports suggested a continuous activity of this reporter throughout larval development [36], our observations indicated its unresponsiveness to damage after 35–39 h (Fig. 4J-L’’’ and Additional File 2: Fig. S2).

Our experiments using IR to induce apoptosis (Additional File 3: Fig. S3) yielded similar results. We observed JNK activation in response to irradiation, regardless of whether it occurred during early stages, when cell proliferation is activated, or late stages, when it is not (Additional File 3: Fig. S3).

The activation of the *DRWNT-GFP* reporter after damage suggests that *wg* might be upregulated in these wings. To investigate this, we examined the expression of a GFP-tagged form of Wingless (Wg-GFP) expressed under the endogenous regulatory region [39] following induction of genetic ablation during pupal development. In the control pupal wing Wg-GFP is consistently expressed along the wing margin during pupal development (Additional File 4: Fig. S4). In the majority of *hh* > *rpr* pupal wings subjected to the same genetic ablation protocol as described in Fig. 3A, the posterior compartment is eliminated (Additional File 4: Fig. S4). Interestingly, at 20-24 h (12 h at 17 °C + 12 h at 29 °C), and 25-29 h (20 h at 29 °C) APF Wg-GFP is expressed at the wound edge in the anterior/posterior border (yellow arrows in Additional File 4: Fig. S4 B’’’, D’’ and D’’’). At 20-24 h (12 h at 17 °C + 12 h at 29 °C) APF a distinct band of cells shows robust expression of Wg-GFP, whereas at 25-29 h APF (20 h at 29 °C) this expression fades and only a few cells at the border express low levels of Wg-GFP (yellow arrows in Additional File 4: Fig. S4 D’’-D’’’). At 30-34 h (12 h at 17 °C + 20 h at 29 °C) APF, Wg-GFP expression is no longer detectable at the wound edge. At later stages (35-39 h APF (20 h at 17 °C + 20 h at 29 °C)), Wg-GFP is not expressed at the wound edge, but its expression is maintained in pupal wings that retain the posterior wing margin (Additional File 4: Fig. S4G-H’’’). These results are consistent with our observation that the *DRWNT-GFP* reporter is activated at the wound edge at 20-24 h (12 h at 17 °C + 12 h at 29 °C), and 25-29 h (20 h at 29 °C) APF. However, they differ from our previous observation of *DRWNT-GFP* reporter activation at 30-34 h (12 h at 17 °C + 20 h at 29 °C) APF. This discrepancy may be due to the persistence of GFP expression in *DRWNT*. Thus, although the reporter could not be activated at that time, GFP expressed at earlier stages may still be present. Collectively, these results suggest that damage results in detectable levels of Wg expression at the wound edge up to 25-29 h (20 h at 29 °C) APF.

We next examined the expression of *dpp* after damage induction using a *dpp-lac Z* reporter. In the control pupal wing, this reporter is expressed in a band of cells at the anterior/posterior border (Additional File 5: Fig. S5 A, C, and E). We found that in damaged wings, the expression pattern closely resembles that observed in control wings. We did not observe ectopic expression in the anterior compartment and the width of the band expressing *dpp-LacZ* in damaged wings is comparable to that in control wings (Additional File 5: Fig. S5 B-B’’’, D-D’’’ F-F’’’ and G). However, we cannot exclude the possibility that the level of *dpp* expression within the band of cells expressing this gene at the anterior/posterior border may be increased in damaged pupal wings. This is because any changes in *dpp* expression resulting from apoptosis in this region could potentially be masked by the normal band of *dpp* expression.

### Down-regulation of JNK signalling reduces the proliferative response

The persistent activity of JNK signalling, even at stages where damage fails to induce a proliferative response, suggests that the reduced proliferative capacity observed during late stages of pupal development is not primarily due to an inability of cells to activate JNK. To further explore this idea, we performed an experiment in which a constitutively activated form of the JNK kinase, Hep (*hep*^*CA*^) was expressed for 20 h at various intervals during pupal development. In accordance with our previous experiments, we examined the proliferative response at the following intervals: 25-29 h (20 h at 29 °C), 30-34 h (12 h at 17 °C + 20 h at 29 °C), 35–39 h (20 h at 17 °C + 20 h at 29 °C) and 45–49 h (48 h at 17 °C + 20 h at 29 °C) APF. We followed the same temperature shift protocol used in the genetic ablation studies (Fig. 3A).

**Fig. 5:**
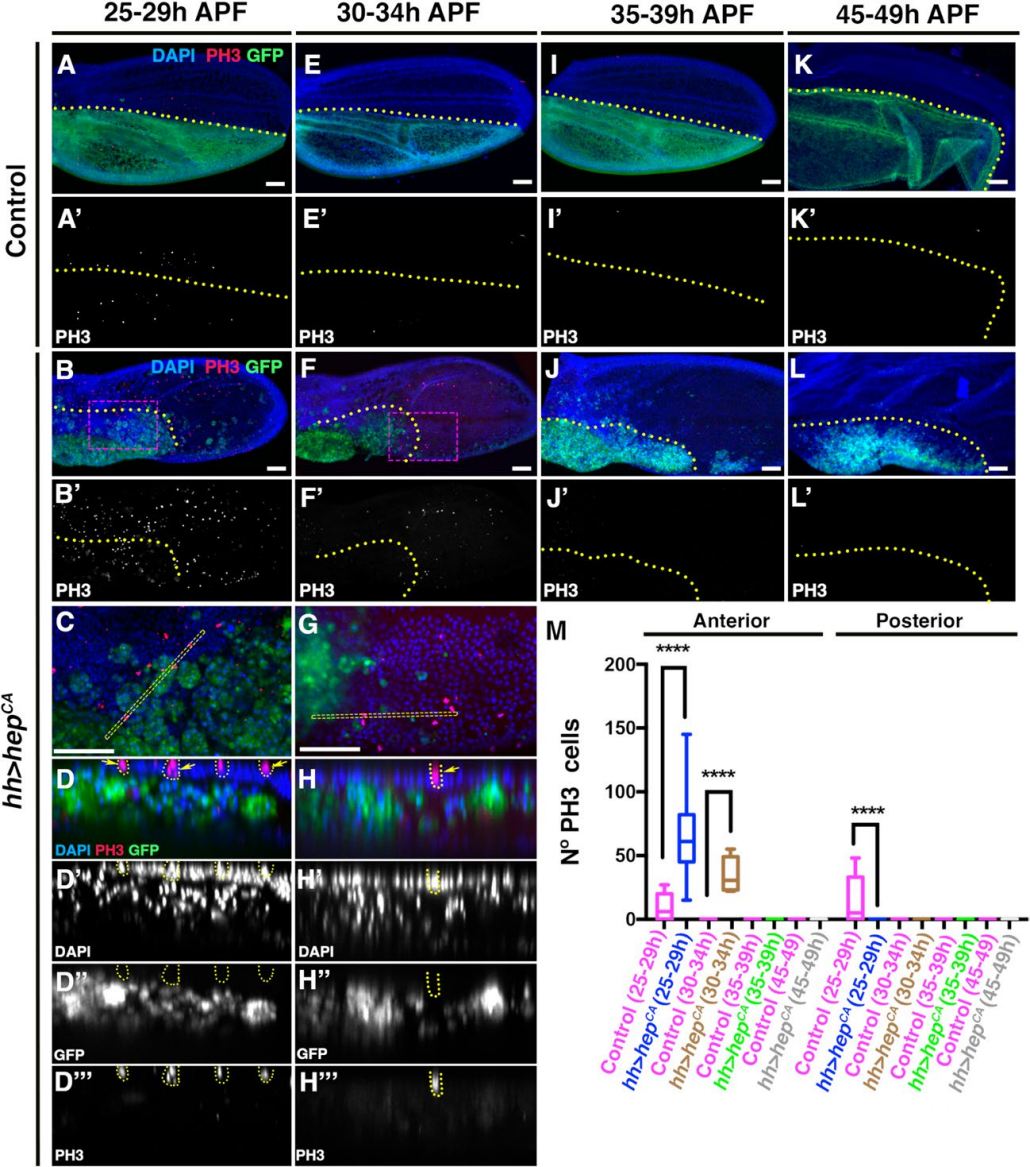
The ectopic activation of *hep*^*CA*^ is not sufficient to expand the proliferative period of pupal wing beyond 30–34 h APF. To establish the developmental times analysed in this experiment, we used the same protocol described in Fig. 3A. For the interval 45-49 h, pupae were subjected to the following temperature shift (48 h at 17 °C + 20 h at 29 °C) (Methods). (A-A’, E-E’, I-I’ and K-K’) *hh-Gal4; tub-Gal80*^ts^ control pupal wings were analyzed at 25–29 h (20 h at 29 °C) (A-A’), 30–34 h (12 h at 17 °C + 20 h at 29 °C) (E-E’), 35–39 h (20 h at 17 °C + 20 h at 29 °C) (I-I’) and at 45–49 h (48 h at 17 °C + 20 h at 29 °C) (K-K’). (B-D’’’, F–H’’’, J-J’, and L-L’) *UAS-hep*^*CA*^*; hh-Gal4 UAS-GFP; tub-Gal80*^*ts*^ pupal wings were examined at developmental stages 25–29 h (20 h at 29 °C) (B-D’’’), 30–34 h (12 h at 17 °C + 20 h at 29 °C) (F–H’’’), 35–39 h (20 h at 17 °C + 20 h at 29 °C) (J-J’) and at 45–49 h (48 h at 17 °C + 20 h at 29 °C) (L-L’). Pupal wings were stained with anti-PH3 (red) DAPI (blue). GFP is shown in green. Images **C**, and **G** correspond to high magnification of the region indicated by a magenta rectangle in images **B** and **F**, respectively. (D-D’’’ and H–H’’’) transverse sections of the pupal wing shown in **B** and **F**, respectively. The location of the sections are indicated by the yellow rectangle in **C** and **G**, respectively. Large aggregates of *hep*^*CA*^-expressing cells are identified (expressing GFP in green) between the two epithelial layers forming the pupal wing. Note that the mitotic cells in the posterior compartment (expressing GFP in green) are not labelled with GFP (yellow arrows in **D** and **H** and outlined by dotted yellow lines in D-D’’’ and H–H’’’) and are positioned apically to the *hep*^*CA*^-expressing cells. **M** The graph shows the number of mitotic cells (PH3 positive) in control wings and in *UAS-hep*^*CA*^*; hh-Gal4 UAS-GFP; tub-Gal80*^*ts*^ wings at different times APF. Error bars represent 99% percentile. Statistical analysis was conducted using multiple comparation t-Test student (Mann-Whitney test) **** *p* < 0.0001. Control (25-29 h) *n* = 15, *hh* > *hep*^*CA*^ (25-29 h) *n* = 11, Control (30-34 h) *n* = 15, *hh* > *hep*^*CA*^ (30-34 h) *n* = 10, Control (35-39 h) *n* = 14, *hh* > *hep*^*CA*^ (35-39 h) *n* = 14, Control (45-49 h) *n* = 10, and *hh* > *hep*^*CA*^ (45-49 h) *n* = 10. Yellow dotted lines indicate the boundary between the anterior and posterior compartments. White scale bar 50 μm

Our results showed that *hep*^*CA*^ expression resulted in a proliferative response only up to 30-34 h (12 h at 17 °C + 20 h at 29 °C) AFP, with no discernible proliferative effect observed at later developmental stages (Fig. 5I-L’ and M). Notably, the induced proliferation occurred in a non-autonomous manner. Cells expressing *hep*^*CA*^ in the posterior compartment, labelled with GFP did not divide (Fig. 5B-H’’’ and M). Although we observe mitotic cells in regions surrounded by GFP-expressing cells (outlined with yellow dotted lines in Fig. 5D-H), these mitotic cells are located apically to the *hep*^*CA*^-expressing cells and do not show GFP expression (Fig. 5C-H’’’). Interestingly, cells expressing *hep*^*CA*^ formed large aggregates that coalesced into a compact mass. Some of these aggregates infiltrated between the two epithelial layers that form the pupal wing (Fig. 5C-H’’’).

These results support our data suggesting that activation of the JNK pathway alone is not sufficient to induce a proliferative response during the later stages of pupal development.

Our observations indicate that the JNK signalling pathway is activated in response to damage during pupal development. To analyze the involvement of this pathway in the induction of compensatory proliferation at this stage, we investigated the effect of blocking JNK signalling in irradiated pupae. To this end, we expressed *bsk*^*DN*^, which encodes a dominant-negative form of the Jun kinase basket [40], under the control of *hh-Gal4*.

**Fig. 6:**
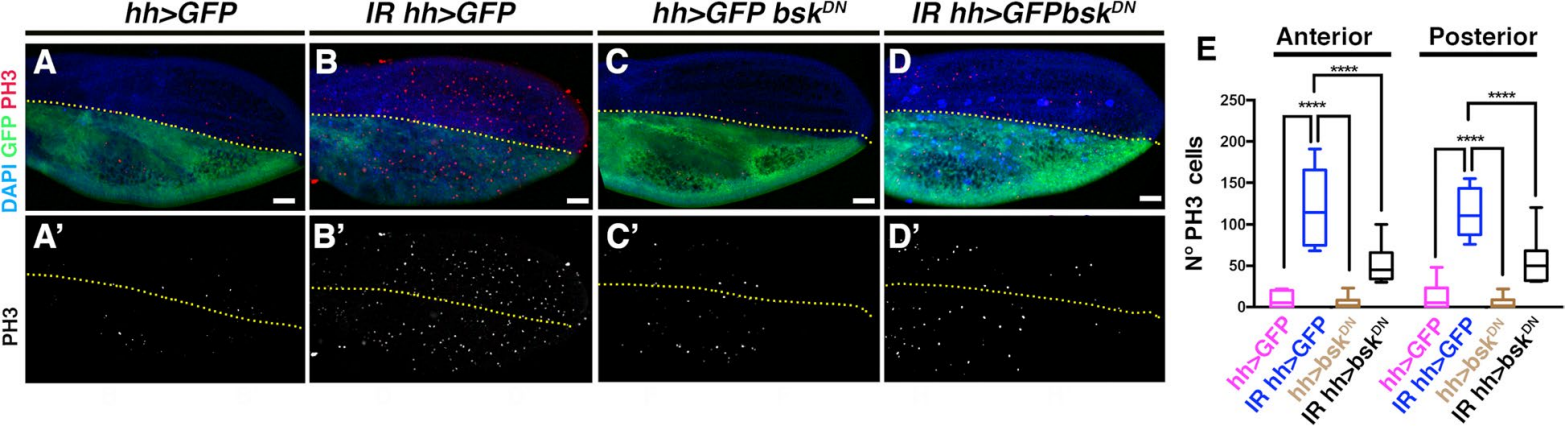
Reduced proliferative response with downregulation of JNK signaling. *UAS-bsk*^*DN*^*; hh-Gal4 tub-Gal80*^*ts*^*/ UAS-GFP* pupae aged 0–4 h were irradiated at 0 h APF for 8 min and immediately transferred to 29 °C for 20 h to activate *bsk*^*DN*^. After this period of time the pupae were dissected. (A-A’) Control: Non-irradiated *hh-Gal4 tub-Gal80*^*ts*^*/ UAS-GFP* pupae kept at 29 °C for 20 h. (B-B’) *hh-Gal4 tub-Gal80*^*ts*^*/ UAS-GFP* pupal wing of pupae irradiated at 0 h APF and transferred to 29 °C for 20 h. (C–C’) *UAS-bsk*^*DN*^*; hh-Gal4 tub-Gal80*^*ts*^*/ UAS-GFP* pupal wing kept at 29 °C for 20 h. (D-D’) *UAS-bsk*^*DN*^*; hh-Gal4 tub-Gal80*^*ts*^*/ UAS-GFP* pupal wing irradiated at 0 h APF and kept at 29 °C for 20 h. Pupal wings were stained with anti-PH3 (red), DAPI (blue) and GFP is shown in green. **E** Graph shows the number of mitotic cells (PH3 positive) in the anterior and posterior compartments of pupal wings: *hh* > *GFP* control: Non-irradiated *hh-Gal4 tub-Gal80ts/ UAS-GFP* pupae kept at 29 °C for 20 h. *IR hh* > *GFP: hh-Gal4 tub-Gal80*^*ts*^*/ UAS-GFP* pupal wing of pupae irradiated at 0 h APF and transferred to 29 °C for 20 h. *hh* > *bsk*^*DN*^: *UAS-bsk*^*DN*^*; hh-Gal4 tub-Gal80*^*ts*^*/ UAS-GFP* pupae kept at 29 °C for 20 h. *IR hh* > *bsk*^*DN*^: irradiated *UAS-bsk*^*DN*^*; hh-Gal4 tub-Gal80*^*ts*^/ *UAS-GFP* pupae irradiated at 0 h APF and kept at 29 °C for 20 h. Statistical analysis was conducted using 2 ways Anova multiple comparation **** *p* < 0.0001. *hh* > *GFP* control *n* = 14, IR *hh* > *GFP n* = 10. *hh* > *bsk*^*DN*^* n* = 12 and IR *hh* > *bsk*^*DN*^* n* = 15. Yellow dotted lines indicate the boundary between the anterior and posterior compartments. White scale bar 50 μm

Pupae 0–4 h APF old were selected and subjected to irradiation. Following irradiation, they were immediately transferred to 29ºC to induce *bsk*^*DN*^ expression for a period of 20 h. After this interval, the pupae were dissected to assess the proliferative response. Our observations revealed a significant reduction in the number of mitotic cells in irradiated *hh* > *bsk*^*DN*^ pupal wings compared to irradiated control wings subjected to an identical temperature shift (Fig. 6). Unexpectedly, this reduction affected both the anterior and posterior compartments. Considering that *bsk*^*DN*^ was only overexpressed in the posterior compartment, we expected a more pronounced effect in this area. Overexpression of *bsk*^*DN*^ in non-irradiated wings had no detectable effect (Fig. 6E).

These results strongly implicate JNK signalling in mediating the proliferative response, and that this effect can have a non-autonomous effect. One of the candidates to be mediating this effect is Wg, that as we have shown is ectopically expressed in damaged wing. We have examined the function of *wg* in inducing the proliferative response during pupal development by blocking its function in irradiated pupae. To this end, we used RNAi against *wg* (*UAS-wgRNAi*) driven by *hh-Gal4*. Using the same timeframe as for JNK reduction, we irradiate pupae at 0–4 h APF and immediately transferred them to 29ºC to induce *UAS-wgRNAi* expression for 20 h. Pupae were then dissected to assess the proliferative response. No significant change in the number of mitotic cells was observed in irradiated *hh* > *wgRNAi* pupal wings compared to irradiated control wings subjected to an identical temperature shift (Additional File 6: Fig. S6). These results suggest that *wg* alone may not be essential for promoting the regenerative response in pupae. Previous reports have shown that compensatory proliferation can occur in the absence of *wg*, making the role of *wg* in promoting proliferative responses complex and context dependent [34, 35, 41].

In conclusion, our results strongly support the necessity of JNK signalling to promote the proliferative response during pupal development. However, elucidating the exact mechanisms is challenging as the observed effects are not exclusively autonomous. Thus, the potential contribution of a systemic function to these results cannot be excluded.

### The over-expression of CycE or E2F1 in damaged pupal wing is not sufficient to promote a proliferative response after 35–40 h APF

**Fig. 7:**
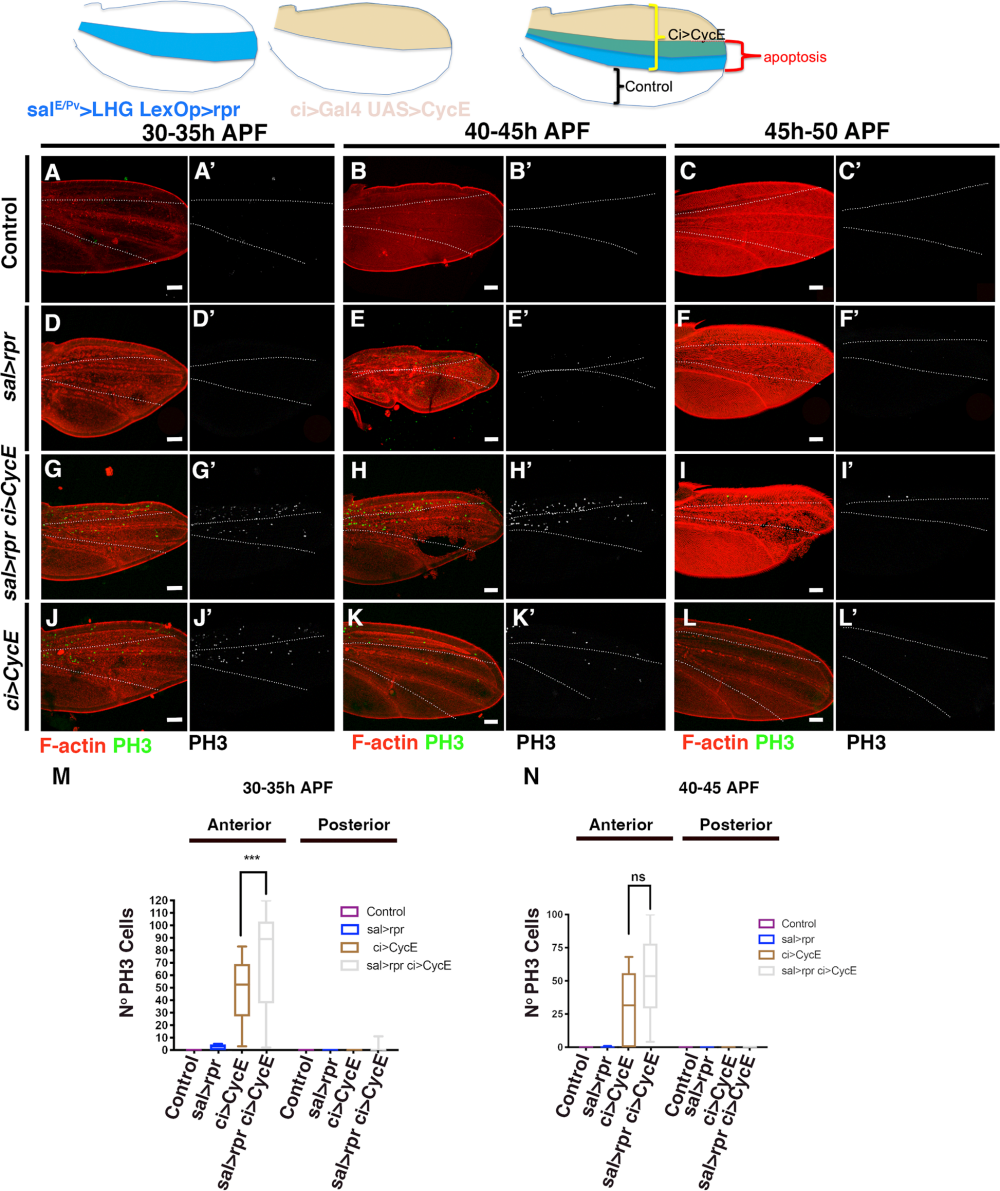
Cyc E is not a limiting factor for apoptosis- induced proliferation during pupal development. To overexpress *rpr* and *Cyc E* in *lexOp-rpr; ci-Gal4; sal*^*E/Pv*^*-LHG tub-Gal80*^*ts*^ pupal wings, pupae were collected at 5-h intervals for staging as described in Methods and then subjected to the following temperature shifts: Developmental stages at 25 °C corresponding to 30–35 h APF: (35 h at 17ºC + 16 h at 29ºC). Development stages at 25 °C corresponding to 40-45 h: (48 h at 17ºC + 16 h at 29ºC). Development stages at 25 °C corresponding to 45–50 h: (60 h at 17ºC + 16 h at 29ºC). (A-C’) Control pupal wings at 30–35 h (35 h at 17ºC + 16 h at 29ºC (A-A’), 40–45 h (48 h at 17ºC + 16 h at 29ºC) (B-B’) and 45–50 h APF (60 h at 17ºC + 16 h at 29ºC) (C–C'). (D-F’) *lexOp-rpr; ci-Gal4; sal*^*E/Pv*^*-LHG tub-Gal80*^*ts*^ pupal wings at 30–35 h (35 h at 17ºC + 16 h at 29ºC (D-D’), 40–45 h (48 h at 17ºC + 16 h at 29ºC) (E-E’) and 45–50 h APF (60 h at 17ºC + 16 h at 29ºC) (F-F'). (G-I’) *lexOp-rpr; ci-Gal4;sal*^*E/Pv*^*-LHG tub-Gal80*^*ts*^* UAS-CycE* pupal wings at 30–35 h (35 h at 17ºC + 16 h at 29ºC (G-G’), 40–45 h (48 h at 17ºC + 16 h at 29ºC) (H–H’) and 45–50 h APF (60 h at 17ºC + 16 h at 29ºC) (I-I'). (J-L’) *ci-Gal4 tub-Gal80*^*ts*^* UAS-CycE* pupal wings at 30–35 h (35 h at 17ºC + 16 h at 29ºC (J-J’), 40–45 h (48 h at 17ºC + 16 h at 29ºC) (K-K’) and 45–50 h APF (60 h at 17ºC + 16 h at 29ºC) (L-L'). (M–N) The graphs show the number of mitotic cells (PH3 positive) in the different experimental condition analysed**.** The pupal wings were stained with anti-PH3 antibody (Green) and Phalloidin to reveal F-Actin (in red). Statistical analysis was conducted using multiple comparation t-Test student (Mann–Whitney test) *** *p* < 0.001. **M** 30-35 h Anterior compartment: *sal* > *rpr n* = 5, *sal* > *rpr ci* > *CycE n* = 9, *ci* > *CycE n* = 10. Posterior compartment *sal* > *rpr n* = 5, *sal* > *rpr ci* > *CycE n* = 9, *ci* > *CycE n* = 10. (N) 40-45 h Anterior compartment: *sal* > *rpr n* = 7, *sal* > *rpr ci* > *CycE n* = 10, *ci* > *CycE n* = 10. Posterior compartment *sal* > *rpr n* = 7, *sal* > *rpr ci* > *CycE n* = 10, *ci* > *CycE n* = 10. The expression domain of *sal* is outlined by the white dotted line. White scale bar 50 μm

Previous studies have shown that while cell proliferation ceases at 20–24 h during normal pupal development, ectopic expression of G1-S transition factors such as CycE/Cdk2, CycD/Cdk4 or E2F1 can prolong the proliferative stage until 36 h APF, but not beyond [11]. This effect has been attributed to the existence of a positive feedback mechanism between CycE and E2F1 [11], whereby the activation of one factor stimulates the function of the other, thereby promoting proliferation. Consequently, when this feedback loop stops at 36 h APF, only the overexpression of both factors can effectively induce proliferation. Our results suggest that damage can similarly prolong proliferation until approximately 34 h APF, consistent with the effect described previously. A plausible explanation for our observations is that damage may selectively promote the function of only one of these factors. Therefore, if the feedback mechanism is no longer active, damage alone may not be sufficient to induce proliferation. If this hypothesis is correct, then the combination of damage and overexpression of the limiting factor (CycE or E2F2) should be sufficient to induce proliferation beyond 34 h APF. To test this idea, we utilized the double transcriptional trans-activator system *sal*^*E/Pv*^*-LHG/lexOp* in combination with *Gal4/UAS* to overexpress *E2F1 Dp* or *CycE* while inducing apoptosis. The *sal*^*E/Pv*^*-LHG* transgene contains a *Gal80* suppressible form of *LexA* [42]. To conditionally express both the *sal*^*E/Pv*^*-LHG/lexOp* and *Gal4/UAS* binary systems, we used *tub-Gal80*^*ts*^. We induced apoptosis in the *sal*^*E/Pv*^*-LHG* domain of the pupal wing by using *lexOp-rpr*, while simultaneously overexpressing *UAS-CycE* or *UAS-E2F1* in the overlapping anterior compartment with the *cubitus interruptus* (*ci*)-*Gal4* line (Fig. 7 and Additional file 7: Fig.S7).

We selected pupae within the 0–5 h old APF range and maintained them at 17ºC for varying periods: 35 h, 48 h or 60 h. These time periods correspond to developmental stages that are equivalent to 12 h, 20 h and 24 h APF at 25ºC, respectively. They were then transferred to 29ºC for 16 h to induce both ectopic expression of *UAS-CycE* (*ci-Gal4, UAS-CycE*) and apoptosis (*salE/Pv-LHG/lexOp-rpr*). Pupal wings were analyzed immediately after these incubation times. Given the 5 h selection intervals, the developmental stages equivalents at 25ºC are: 30-35 h (35 h at 17ºC + 16 h at 29ºC), 40-45 h (48 h at 17ºC + 16 h at 29ºC) and 45-50 h (60 h at 17ºC + 16 h at 29 °C) APF.

At the 30-35 h (35 h at 17ºC + 16 h at 29ºC) interval, a significant presence of mitotic cells was observed exclusively in the anterior compartment (*CycE*-expressing cells), but not in the posterior compartment, in both *ci* > *CycE* and *sal* > *rpr ci* > *CycE* pupal wings (Fig. 7G-J’). In *sal* > *rpr ci* > *CycE* wings the number of mitotic cells was significantly higher than in *ci* > *CycE* wings (Fig. 7M). Interestingly, at 40-45 h (48 h at 17ºC + 16 h at 29ºC), the number of mitotic cells in *ci* > *CycE* and *sal* > *rpr ci* > *CycE* wings was comparable and significantly higher than in control wings (Fig. 7B-K’ and N). Previous reports have suggested that *CycE* expression alone may not be sufficient to induce mitosis during this time interval [11], temporal differences due to temperature shifts could potentially account for these discrepancies. No mitotic cells were identified after 45 h APF (60 h at 17ºC + 16 h at 29 °C) in either *CycE*-expressing pupal wings (*n* = 10) or wings co-expressing *CycE* and *rpr* (*n* = 10) (Fig. 7C-L’).

Similar results were obtained when apoptosis was induced at the same time that E2F1 was over-expressed (Additional File 7: Fig. S7). Similar to the observations with *CycE*, at 40-45 h (48 h at 17ºC + 16 h at 29ºC) mitotic cells were still evident (Additional File 7: Fig. S7). At this time point, the number of mitotic cells in both *ci* > *E2F1 Dp* and *sal* > *rpr ci* > *E2F1 Dp* pupal wings was comparable. No mitotic cells were detected in pupal wings older than 45 h (60 h at 17ºC + 16 h at 29 °C) (*ci* > *E2F1 Dp n* = 10 and *sal* > *rpr ci* > *E2F1Dp n* = 9). Taken together, our results strongly suggest that overexpression of *CycE* or *E2F1* alone was not sufficient to induce a proliferative response to damage after 35 h APF.

Next, we examined the proliferative response to irradiation when either *CycE* or *E2F1* was overexpressed in the pupal wing. Both factors were overexpressed in the posterior compartment under the control of *engrailed-Gal4*. Pupae were selected at 0–4 h APF (Methods) and immediately irradiated (IR 0 h). They were then transferred to 29ºC to activate *CycE* or *E2F1*, and then dissected at a developmental stage corresponding to 25–29 h APF at 25 °C. Alternatively, they were irradiated 20 h after transfer to 29 °C (IR 20 h). At 29 °C this corresponds to a development stage of 25 h at 25 °C. After irradiation, pupae were transferred to 29 °C for a further 20 h before dissection at a developmental stage corresponding to 52–56 h APF at 25 °C.

In both cases, pupal wings overexpressing *en* > *CycE* and *en* > *E2F1 Dp* at 25–29 h (20 h at 29ºC) showed multiple mitotic cells in the posterior compartment (Additional File 8: Fig. S8 B-E’, J and K). In *en* > *CycE* pupal wings irradiated at 0 h, there was no significant increase in the number of mitotic cells in either the anterior or posterior compartment compared to non-irradiated *en* > *CycE* wings or irradiated controls (Additional File 8: Fig. S8 J). Unexpectedly, irradiating *en* > *E2F1 Dp*-overexpressing pupal wings at 0 h resulted in more mitotic cells in the anterior compartment than control wings irradiated at 0 h and non-irradiated *en* > *E2F1 Dp* wings. However, in the posterior compartment, irradiation not only failed to increase the number of mitotic cells, but actually decreased it (Additional File 8: Fig. S8 K).

In pupal wings irradiated at 20 h APF (corresponding to developmental stage 25 h at 25 °C) and analysed at 40-44 h APF (corresponding to developmental stage 52–56 h at 25 °C), no mitotic cells were observed in either *en* > *CycE* or *en* > *E2F1*, overexpressing wings (Additional File 8: Fig. S8 F-I’).

**Fig. 8:**
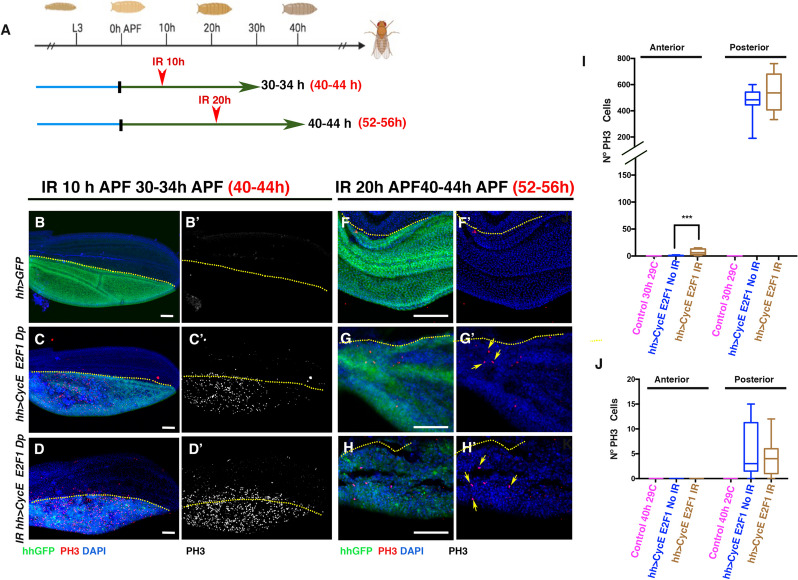
Co-expression of *CycE* and *E2F1* are not sufficient for promoting a proliferative response after damaging in late stages of pupal development. **A** Schematic diagram of the irradiation times employed in this experiment. Selected *hh-Gal4; tub-Gal80*^*ts*^* UAS-CycE UAS-E2F1 UAS-Dp/UAS-GFP* or *hh-Gal4; tub-Gal80*^*ts*^* UAS-GFP* Pupae aged 0–4 h were transferred to 29 °C and irradiated at 10 h APF (IR 10 h) or 20 h APF (IR 20 h) for 8 min and then kept at 29 °C for another 20 h before dissection (20 h after irradiation). The black text indicates the duration of incubation at 29 °C, while the red text indicates the equivalent time at 25 °C. (B-D’) Wings from pupae irradiated at 10 h APF (IR 10 h) and non-irradiated control pupal wings examined at 30–34 h at 29 °C (corresponding to 40–44 h at 25 °C). Non-irradiated *hh-Gal4; UAS-GFP* (B-B’), *hh-Gal4; tub-Gal80*^*ts*^* UAS-CycE UAS-E2F1 UAS-Dp/UAS-GFP* non-irradiated (C–C’), and irradiated *hh-Gal4; UAS-CycE UAS-E2F1 UAS-Dp/UAS-GFP* (D-D’). (F–H’) Wings from pupae incubated during 20 h at 29 °C and then irradiated (IR 20 h) and examine 20 h later or control incubated during 40 h at 29 °C but non-irradiated. Both were maintained at 29 °C for a total duration of 40 h (40–44 h, corresponding to 52–56 h at 25 °C). *hh-Gal4; tub-Gal80*^*ts*^* UAS-GFP* Non-irradiated (F-F’), *hh-Gal4; tub-Gal80*^*ts*^* UAS-CycE UAS-E2F1 UAS-Dp/UAS-GFP non-* irradiated (G-G’), and irradiated *hh-Gal4; tub-Gal80*^*ts*^* UAS-CycE UAS-E2F1 UAS-Dp/UAS-GFP* (H–H’). The wings were stained with anti- PH3 antibody (in red) and DAPI (in blue). *UAS-GFP* is shown in green. Note that pupal wings at 40–44 h, corresponding to stages equivalent to 52–56 h at 25 °C (**G**-**H**), whether irradiated or non-irradiated, show mitotic cells as indicated by the yellow arrows in G' and H'. **I**-**J** The graphs show the number of mitotic cells (PH3-positive) in both the anterior and posterior compartments of the different genetic variants analysed at 30–34 h (corresponding to 40–44 h at 25 °C) (**I**) and at 40–44 h (corresponding to 52–56 h at 25 °C) (**J**). Statistical analysis was conducted using multiple comparation t-Test student (Mann–Whitney test) *** *p* < 0.001. **I** Control *hh* > *GFP* kept 30 h at 29 °C (Control 30 h at 29 °C) *n* = 15, *hh* > *CycE E2F1* No irradiated kept 30 h at 29 °C (*hh* > *cycE E2F1* No IR) *n* = 10, *hh* > *CycE E2F1* irradiated kept 30 h at 29 °C (*hh* > *cycE E2F1* IR) *n* = 9. **J** Control *hh* > *GFP* kept 40 h at 29 °C (Control 40 h at 29 °C) *n* = 15, *hh* > *CycE E2F1* No irradiated kept 40 h at 29 °C (*hh* > *cycE E2F1* No IR) *n* = 6, *hh* > *CycE E2F1* irradiated kept 40 h at 29 °C (*hh* > *cycE E2F1* IR) *n* = 7. White scale bar 50 μm

As mentioned above, there is evidence that co-expression of CycE and E2F1 after 36 h APF can sustain cell proliferation even at very late stages of pupal development [11]. To investigate whether cell cycle reactivation by ectopic expression of these factors enables cells to initiate a proliferative response, we ectopically co-expressed *CycE* and *E2F1* and analyzed the effects of irradiation during pupal stages. Both *CycE* and *E2F1* were co-expressed in the posterior compartment of the pupal wing under the control of *hh-Gal4*. We specifically investigated the effects at late stages, when cells have already exited the cell cycle. Pupae aged 0–4 h were transferred to 29ºC to activate *CycE* and *E2F1* expression after puparium formation. They were irradiated either 10 h (IR 10 h) or 20 h later (IR 20 h). Both groups were analyzed 20 h after irradiation at 30–44 h and 40–44 h at 29ºC (corresponding to developmental stages 40–44 h and 52–56 h at 25 °C, respectively) (see Fig. 8A). Pupal wings co-expressing *hh* > *CycE-E2F1 Dp* at developmental stages equivalent to 40–44 h at 25 °C (30 h at 29ºC) contains a remarkably high number of mitotic cells in the posterior compartment (Fig. 8C–C' and I). Irradiated *hh* > *CycE-E2F1 Dp* pupal wings analysed at the same developmental stages show a similar number of mitotic cells (Fig. 8D-D' and I). However, as previously described, when we ectopically overexpressed only *E2F1 Dp*, there were more mitotic cells in the anterior compartment than in control wings (Fig. 8D-D' and I). In pupal wings irradiated at 20 h APF and analyzed at 40-44 h APF (corresponding to developmental stage 52–56 h at 25 °C), we still find mitotic cells in the posterior compartment (Fig. 8G'), confirming that the co-expression of these factors can induce proliferation at very late stages of pupal development [11]. However, this effect is not further enhanced by irradiation (Fig. 8H–H' and J).

Taken together, these results suggest that although cells can be forced to re-enter the cell cycle by overexpressing cell cycle regulators at very late stages of pupal development, this does not appear to be sufficient to enable them to respond to the mitotic signals produce by apoptotic cells beyond 34 h APF.

### Ecdysone-responsive transcription factor E93 blocks the proliferative response during pupal development

**Fig. 9:**
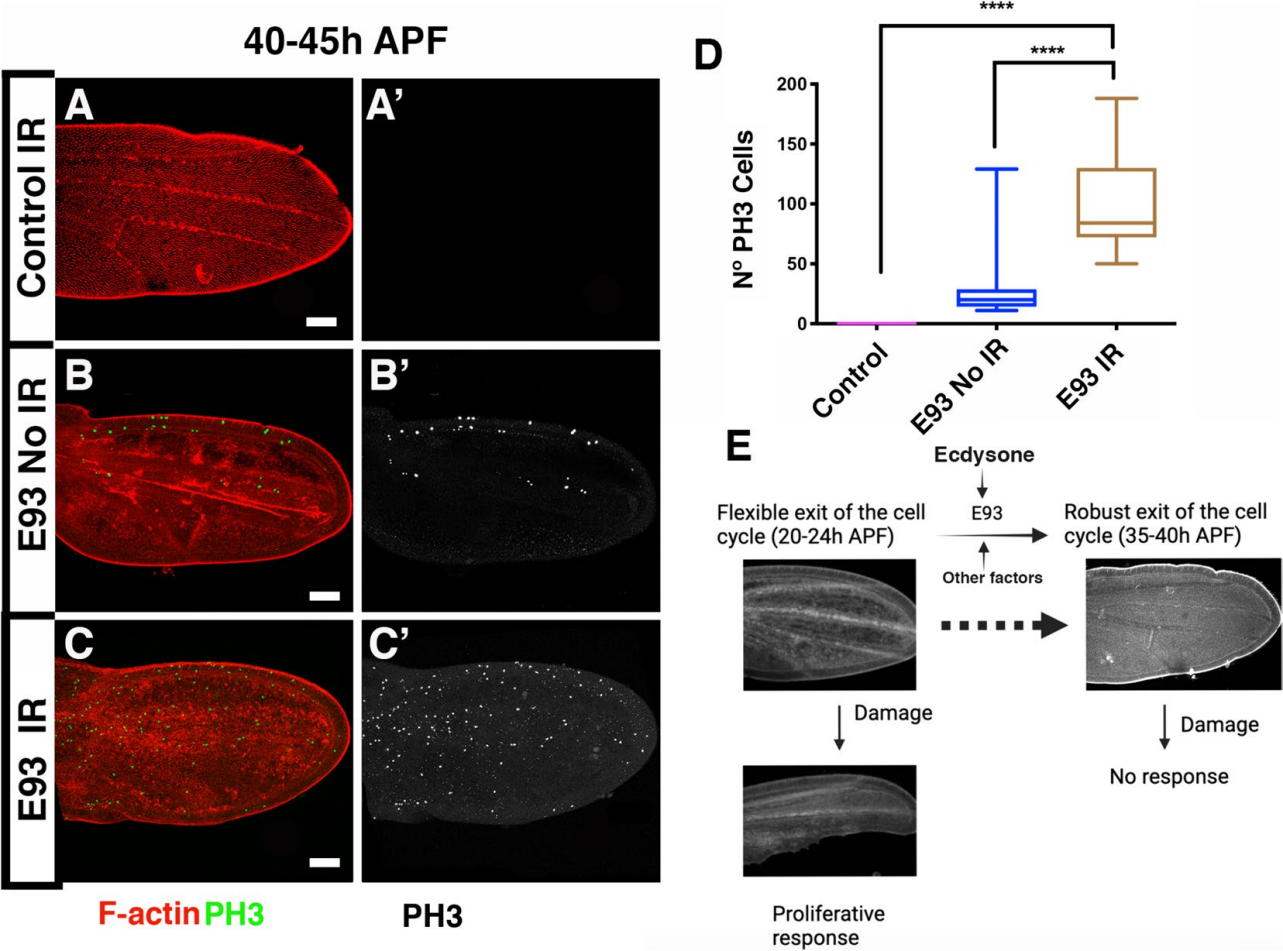
In *E93* mutant damage prolongs proliferative response beyond 40 h APF. Pupae aged 0–5 h were reared at a constant temperature of 25˚C and irradiated 20 h APF. Dissections were performed 20 h after irradiation. Given the 5 h interval chosen, the age of the pupae at the time of analysis would be approximately 40–45 h APF. (A-C’) Pupal wings at 40–45 h APF. Control (A-A’), *E93* non-irradiated pupal wings (B-B’) and *E93* irradiated pupal wings (C–C'). **D** The graphs show the number of mitotic cells (PH3 positive) in the different experimental condition analyzed**.** The pupal wings were stained with anti- PH3 antibody (in Green A-C, and grey A’-C’) and Phalloidin to reveal F-Actin (in red A-C). **E** A proposed model for the dynamic response to damage during pupal development. Statistical analysis was conducted using One-way ANOVA Tukey's test **** *p* < 0.0001. Control *n* = 12, *E93* non-irradiated *n* = 10, *E93* irradiated *n* = 11. White scale bar 50 μm

The end of the positive feedback loop between CycE and E2F1, as well as the ending of the proliferative response following damage, coincide with the initiation of epigenetic silencing of the regulatory regions of critical genes involved in cell cycle regulation, including *CycE*, *E2F1*, and *stg* [14, 17, 18]. In *Drosophila*, developmental transitions are regulated by the hormone ecdysone, and ecdysone-responsive transcription factors control temporal changes in chromatin accessibility during pupal wing development [14, 17, 18]. Specifically, the E93 transcription factor is transcriptionally activated at 18 h and 24 h APF [18]. Loss-of-function mutations in the *E93* gene results in chromatin accessibility changes at several genome regions in 24 h and 44 h APF pupal wings [18]. Importantly, the progressively closed chromatin status observed at regulatory regions of the *CycE*, *E2F1* and *stg* genes between third instar and 44 h APF is attenuated in *E93* mutants [18] (Additional File 9: Fig. S9). Given that this temporal window coincides with the end of apoptosis-induced proliferation during pupal development, it is plausible that E93, by blocking the chromatin accessibility at these cell cycle regulators, could help prevent the induction of this response. To explore this idea, we have irradiated *E93* mutant pupae at 20 h APF and examined the proliferative response 20 h later (40 h APF). In non-irradiated *E93* mutant pupal wings, we still observed some PH3 positive cells at 40–45 h APF. Interestingly, there is an increase in the number of mitotic cells in the irradiated *E93* mutant pupae (Fig. 9A-D). This finding highlights the importance of E93 in regulating cell proliferation during pupal wing development.

Our results strongly suggest the involvement of E93 in the intricate cellular processes that regulate and limit the proliferative response of pupal cells towards the end of their development. However, it should be noted that these results do not exclude the possibility that other factors may also play a fundamental role in limiting the proliferative response of pupal cells.

## Discussion

Regeneration is a complex process that relies on multiple cellular and molecular mechanisms working in concert to uphold tissue homeostasis and effectively respond to external challenges. One critical aspect of this process is the induction of regenerative growth, which is often lost during development or aging. In *Drosophila* development, multiple studies have shown that cell proliferation is one of the primary responses during disc regeneration in larval stages [8, 43–48]. When damage occurs, apoptotic cells generated by the insult can initiate a process known as Apoptosis-induced proliferation [32, 49]. Different studies using third instar larval imaginal discs have suggested that this ability is lost at the end of larval stage or during pupal development [9, 10]. However, our work shows that damage induction in the pupal wings produces a proliferative response that is maintained up to 34 h APF.

Previous studies have demonstrated that overexpression of key regulators of the G1-S transition, such as CycE/Cdk2, CycD/Cdk4, or E2F1, in pupal stages can maintain cell division until 36 h APF. However, overexpression of these regulators after that time is not sufficient to activate cell proliferation. To keep cells proliferating until at least 40–44 h APF, ectopic activation of both CycE and E2F1 is necessary. It has been proposed that this is due to the existence of a positive feedback mechanism between CycE and E2F that is active during larval and early pupal development, but finishes after 36 h APF [11, 14, 17]. Our findings reveal that induction of apoptosis in pupal wings before 24 h APF induces cell proliferation up to 34 h APF, which coincides with the end of the positive feedback between CycE and E2F1. This observation suggests that damage could trigger proliferation-promoting signals through one of these factors. Consequently, a combination of damage and activation of one of these cell cycle regulators should theoretically induce proliferation after 36 h. However, our data suggest that this is not the case. The results presented here suggest that beyond 36 h, ectopic expression of cell cycle regulators, even when two of them (CycE and E2F1) are co-expressed, is not sufficient to counteract the signals that drive cell cycle exit at the end of the proliferative phase and subsequently allow a proliferative response to damage. It has been suggested that ectopic expression of cell cycle regulator acts through accessible regulatory sites to 'short-circuit' the robust G0 exit that occurs at 36 h, this reactivate *CycE* and *stg* and thereby reinitiate the cell cycle [17]. However, this alone does not appear to be sufficient to make cells responsive to the mitotic signals induced by apoptotic cells.

A surprising finding is that overexpression of *E2F1 Dp* can non-autonomously induce neighbouring cells to respond to proliferative stimuli. We have also observed other non-autonomous effects; damage in the posterior compartment can induce proliferation at relatively long distances. Similarly, ectopic expression of *hep*^*CA*^ also promotes cell proliferation far from where its activation is induced. Part of these effects could possibly be attributed to the phenomenon of apoptosis-induced apoptosis [49, 50]. Apoptotic cells release death factors that promote apoptosis in neighbouring cells. Indeed, we have found that the induction of cell death in the posterior compartment increases apoptosis throughout the anterior compartment (Fig. 3C). This non-autonomous death can generate new proliferative signals that affect surrounding cells. In addition, factors such as reactive oxygen species released by apoptotic cells [51] can diffuse over long distances and mediate non-autonomous effects. Finally, we cannot exclude the possibility that damage may have a systemic effect leading to changes in the proliferation pattern.

### E93 is involved in blocking apoptosis-induce proliferation

In *Drosophila* developmental transitions are regulated by the hormone Ecdysone. The signals induce by this hormone are mediated by the ecdysone-responsive transcription factors that are involved in regulating temporal changes in chromatin accessibility that occur throughout wing disc development [14, 17, 18]. Specifically, the E93 transcription factor is expressed at 18 h and 24 h APF [18]. Mutation of the *E93* gene results not only in the loss of accessibility in several regions of the genome, but also in the aberrant presence of numerous regions of open chromatin at 24 and 44 h APF pupal wings, including regulatory regions adjacent to the cell cycle regulator *CycE*, *E2F1* and *stg* (Additional File 9: Fig. S9). Therefore, E93 during pupal development seems to be involved in participating in the irreversible withdrawal of pupal wing cells from the cell cycle by contributing to the epigenetic silencing of regulatory regions in essential cell cycle regulator genes.

During pupal development, the induction of cell death triggers the activation of JNK pathway, both in early [19] and late stages, even when apoptosis-induced proliferation is not observed. Although the JNK pathway is known to suppress the Polycomb group of proteins [22], our results suggest that its activation in pupal wings is not enough to overcome epigenetic silencing of key cell cycle regulators.

Our data suggest that alterations in chromatin accessibility at 36 h APF, influenced by ecdysone-mediated developmental programming and possibly other factors [14, 17, 18], result in a robust exit from the cell cycle. In addition, these changes in chromatin accessibility are likely to affect other essential genes required for the initiation of proliferative regeneration. Consequently, at 36 h APF, wing cells become unresponsive to damage-induced signals, preventing the initiation of regenerative proliferation (Fig. 9E). This model is likely to be applicable to other organisms, such as urodeles, zebrafish, and mice. For instance, Lin-28, an RNA-binding protein that regulates Let-7, has been shown to inhibit the expression of thyroid hormone target genes, delay development, and prolong regenerative potential after damage in urodeles [52]. Similarly, inhibiting thyroid hormone signaling at the level of its synthesis and at the receptor level in neonatal mice rescues the proliferative capacity of cardiomyocytes and the regenerative potential of the adult heart [53]. Conversely, exogenous administration of thyroid hormones in zebrafish inhibits the regenerative capacity of the heart and caudal fin [54]. All these findings suggest that hormones play an important role in regulating the proliferative capacity of specific tissues in vertebrates, potentially serving as essential factors in limiting regenerative capacity during vertebrate development.

The result presented in this work highlights the intricate interplay between developmental cues, chromatin modifications, and the regulation of cell cycle dynamics, shedding light on the mechanisms underlying the fine-tuned control of cellular proliferation and regeneration.

## Conclusions

Our investigation of the proliferative response triggered by cell death during pupal development has shown that apoptosis induction prolongs the proliferative phase up to 34 h APF in wing cells, after which cell death alone is no longer sufficient to induce proliferation. In addition, we found that the reactivation of the cell cycle by the ectopic co-expression of cell cycle regulators (CycE and E2F1) is not sufficient to counteract the signals that drive the exit from the cell cycle at the end of the proliferative phase to allow a proliferative response to damage. Importantly, we show that the inability to reactivate the cell cycle and induce a proliferative response is not due to a failure to activate the JNK pathway—a signal fundamental to regenerative proliferation. Instead, our data highlight the critical role of the ecdysone-responsive transcription factor E93 in regulating and limiting the proliferative response during pupal development.

## Methods

### Fly strain

The following strains were used in this study. Unless otherwise was indicated, strain descriptions can be found at http://flybase.bio.indiana.edu.

*w; en-Gal4 UAS-GFP; tub-Gal80*^*ts*^*,* (*en-Gal4*, Flybase ID: FBti0003572) *w; tub-Gal80*^*ts*^*; hh-Gal4* (*hh-Gal4* [55]) *(tub-Gal80*^*ts*^*,* described in Flybase, Bloomington Drosophila Stock Centre*), w; ci-Gal4; sal*^*E/Pv*^* LHG, tub-Gal80*^*ts*^ [51]*, UAS-CycE(II)* (BDSC#30924)*, UAS-reaper* (BDSC#5823)*, UAS-E2F1, UAS-Dp* (BDSC#4770)*, UAS-wgRNAi (v13351,* VDRC*), lexOp-rpr* [51]*, TRE-DsRed* [38]*, UAS-hep*^*CA*^, *UAS-bsk*^*DN*^ [40] (BDSC#6409), *DRWNT-GFP* reporter [36]*, P{Tub-DBS-S-H2Av-GFP}II* (BDSC#83129), *dpp-LacZ* (p10638, BDSC#12379), and *Wg-GFP* [39].

### Protocol irradiation experiments

To determine the age of the pupae for the different experiments, we started the process by transferring late third instar larvae to new containers. After a period of 2, or 4 h, we carefully selected the pupae. Therefore, these pupae will be: 0–2 h, or 0–4 h old. These selected pupae were then allowed to develop further at 25 °C to reach the desired age for each specific experiment. They were irradiated during 8 min at (0 h APF), 5 h later (5 h APF hours), 10 h (10 h APF), 15 h (15 h APF) and 20 h (20 h APF). The pupal wings were analysed 20 h after irradiation.

### Variation in the rate of development at different temperatures

To study the rate of development at different temperatures, we established three sets of pupae between 0–4 h old and reared them simultaneously at different temperatures: 17 °C, 25 °C and 29 °C. Our results showed that at 25 °C the pupae hatched after 100 ± 5 h, whereas at 17 °C and 29 °C they hatched after 245 ± 7 and 79 ± 3 h respectively. Therefore, we found that pupal development was 2.5 times slower at 17˚C compared to 25˚C and 1.3 times faster at 29˚C compared to 25˚C under our experimental conditions.

### Protocol for genetic ablation experiments

*UAS-rpr; hh-Gal4 tub-Gal80*^*ts*^*/* + larvae were grown at 17 °C and then aged at the pupal stage according to the above protocol. Pupae were maintained at 17 °C for varying lengths of time before being transferred to 29 °C to activate *rpr* expression for 20 h (except for the 20–24 h interval, see below).

Considering the different temperature conditions and the observed developmental rate discrepancies (2.5 times slower at 17 °C and 1.3 times faster at 29 °C), we adjusted the duration of pupal stages at 17 °C to align with the developmental stages observed at 25 °C, ensuring consistency.

Given our selection intervals of 4 h and the defined differences in developmental rates (development is 2.5 times slower at 17 °C and 1.3 times faster at 29 °C), we assumed that developmental stages corresponding to 20–24 h, 25–29 h, 30–34 h and 35–39 h APF at 25 °C would be generated following the subsequent temperature shifts:

- Developmental stages corresponding to 20–24 h at 25 °C: (12 h at 17 °C) + (12 h at 29 °C).
- Developmental stages corresponding to 25–29 h at 25 °C: (20 h at 29 °C).
- Developmental stages corresponding to 30–34 h at 25 °C: (12 h at 17 °C) + (20 h at 29 °C).
- Developmental stages corresponding to 35–39 h at 25 °C: (20 h at 17 °C) + (20 h at 29 °C).
- Developmental stages corresponding to 45–49 h at 25 °C: (48 h at 17 °C) + (20 h at 29 °C).

Pupae were fixed at the desired ages.

To account for developmental differences, we minimized the duration of exposure to 29 °C during the interval (20–24 h). This adjustment was necessary because 20 h at this temperature would result in a developmental stage at 25 °C that exceeds 24 h.

### The temperature shift protocol used in Fig. 7 and Additional File 7: Fig. S7

Pupae were collected at 5 h intervals for staging as mentioned above, followed by the application of the following temperature shifts:

- Developmental stages at 25 °C, 30-35 h: (35 h at 17ºC) + (16 h at 29ºC).
- Developmental stages at 25 °C, 40-45 h: (48 h at 17ºC) + (16 h at 29ºC).
- Developmental stages at 25 °C 45-50 h: (60 h at 17ºC) + (16 h at 29 °C).

### Temperature shift protocol used in Fig. 8 and Additional file 1: Fig.S8

To study the effect of ectopic expression of the different *UAS* lines used in our analysis, *en-Gal4 tub-Gal80*^*ts*^* UAS-X* or *hh-Gal4 tub-Gal80*^*ts*^* UAS-X* larvae (where X indicates the different transgenes used in our assay) were reared at 17ºC and then collected at 4-h intervals for staging as described above. Irradiation was performed at three different times:

- IR0h: Pupae were irradiated for 8 min at 0 h APF and then transferred to 29 °C for 20 h. Dissection was performed at developmental stages at 25 °C corresponding to 25–29 h APF.
- IR10h: Pupae were transferred to 29 °C at 0–4 h APF and 10 h later irradiated for 8 min. After irradiation, pupae were transferred to 29 °C for a further 20 h before dissection at a developmental stage corresponding to 40–44 h APF at 25 °C.
- IR20h: Pupae were transferred to 29 °C at 0–4 h APF and 20 h later irradiated for 8 min (developmental stages at 25 °C corresponding to 25–29 h APF). After irradiation, pupae were transferred to 29 °C for a further 20 h before dissection at a developmental stage corresponding to 52–56 h APF at 25 °C.

### Irradiation

Pupae were given a dose of 4000 R using Philips-MG-102 irradiation unit.

### Quantitative analysis

The number of mitotic cells was determined by counting the total number of cells expressing the mitotic marker phospho-histone 3 over the entire wing blade. In some experiments, we indicate the mitotic cells present in the anterior and posterior compartments. In the experiment shown in Fig. 5, where *hep*^*CA*^ was ectopically expressed, the number of mitotic cells in the anterior compartment (not expressing GFP) was assessed by counting the total number of cells expressing PH3 in this region. For the posterior compartment (cells expressing *UA*S-*hep*^*CA*^), only those mitotic cells that co-expressed both, the mitotic marker PH3 and GFP were considered.

For the apoptotic analysis, images were processed using ImageJ software (NUH, Bethesda, USA). Each image analysed was a representative section of the wing. For each image, an intensity threshold was set corresponding to the labelling with the antibody Dcp-1 or the expression of DBS histone-GFP in the nuclei. A region of interest (ROI) was then generated. For Dcp-1 we selected the entire wing surface, for DBS-Histone-GFP we selected the region between vein 5 and the wing margin to avoid the presence of presumptive hemocytes. We then calculated the percentage of the ROI covered by the staining using the area fraction option in set measurements.

To analyse the % *TRE-GFP* expression, as shown in Additional File 3: Fig. S3 I, we followed a procedure similar to the analysis of apoptotic cells. Images were processed using ImageJ software (NIH, Bethesda, USA). For each image, an intensity threshold was set corresponding to the *TRE-GFP* signal in the wing epithelial cells. A ROI was then generated, selecting the area between vein 4 and 5 to exclude the presence of presumptive hemocytes. The percentage of the ROI covered by *TRE-GFP* signal was then calculated.

Statistical analysis was performed using Graph Pad Prism software (https://www.graphpad.com). The specific statistical test and the n used in each analysis are noted in the corresponding figure.

### Immunocytochemistry

Immunostaining of the pupal wings was performed according to [56]. The following primary antibodies were used: rabbit and mouse anti-pH3 1/500 (Merck Millipore #06–570 and Cell signal technology #9796), rabbit anti-cleaved Dcp1 1:200 (Cell Signalling Technology #95785), and anti-ß Galactosidase 1:500 (MP Biomedics #559761 and Promega #Z378A, 1:1000). To stain cellular F-actin we used Phalloidin-TRITC (Sigma-Aldrich Cat#P1951) (1:200), Phalloidin-Alexa 488 (ThermoFisher A-12379) and Phalloidin-Alexa 555 (ThermoFisher A-34055) (1:200). To stain nuclei we used DAPI Merck (Ref268298).

Secondary antibodies (ThermoFisher) were used at dilutions of 1:200.

Pupal wings were mounted in Vectashield mounting fluorescent medium (Vector Laboratories, Inc. REF H-1000).

### Supplementary Information

**Additional file 1:** **Fig. S1.** Proliferative and apoptotic response in the pupal wing induced by X-ray irradiation at different times after puparium formation Selected 0-4h old pupae were irradiated for 8 minutes at 15h (IR15h APF) and 20h (IR20h APF) (see Fig. 1A). Pupal wings were analyzed 20h after exposure (35-39h) and (40-44h) APF. (A-A’’’’, and C-C’’’’) P{Tub-DBS-S-H2Av-GFP control pupal wing at 35-39 hours (A-A’’’’), and 40-44 hours (C-C’’’’) APF. (B-B’’’’, and DD’’’’) P{Tub-DBS-S-H2Av-GFP pupal wing irradiated at 15h IR APF (IR 15h) and analyzed at 35-39 hours (B-B’’’’), and at 20 h APF (IR 20h) and analyzed at 40- 44 hours APF (D-D’’’’). In non-apoptotic cells, DBS-GFP is expressed in the membranes of the cells. Images A’’’-A’’’’, B’’’-B’’’’, C’’’-C’’’’ and D’’’-D’’’’ correspond to high magnification of the region indicated by a magenta rectangle in images A-D. White scale bar 50μm.

**Additional file 2:** **Fig. S2.** Patterns of JNK and BRV activation following genetic ablation of the posterior compartment in the pupal wing. To analyze development stages corresponding to 45-49 h APF at 25°C, *UAS-rpr; DRWNT-GFP hh-Gal4; tub-Gal80*^ts^ *TRE-RFP* pupae were kept at 17°C during 48h APF and then transferred to 29°C for a period of 20h. (A-A’’) *DRWNT-GFP* *hh-Gal; tub-Gal80*^*ts*^ *TRE-RFP* control pupal wings at 45-49h APF. (B-B’’) *UAS*-*rpr; DRWNT-GFP hh-Gal4; tub-Gal80*^*ts*^ pupal wing after over-expressing rpr during 20 h (48h at 17°C and 20h at 29°C). White scale bar 50μm.

**Additional file 3:** **Fig. S3.** Pattern of JNK activation following irradiation in pupal wing disc. Selected 0-4 h old TRE-GFP pupae were irradiated for 8 minutes at 0 h APF (IR 0h APF), 5 hours later (IR 5h APF hours), at 10 h APF (IR 10h APF), and at 20 h APF (IR 20h APF). They have been maintained at 25°C for the entire development period. Pupal wings were analyzed 20 h after exposure. (A-A’’’’, CC’’’’, E-E’’’’ and G-G’’) TRE-GFP control non-irradiated pupal wing at 20-24 hours (A-A’’’’), 25-29 hours (C-C’’’’) 30-34 hours (E-E’’’’) and 40-44 hours APF (G-G’’). (B-B’’’’, D-D’’’’, F-F’’’’ and H-H’’) TRE-GFP pupal wing irradiated at 0 h APF and analyzed at 20-24 hours (B-B’’’’), irradiated at 5h APF and analyzed at 25-29 hours (D-D’’’’), irradiated at 10 h APF and analyzed at 30-34 hours APF (F-F’’’’), and irradiated at 20 h APF and analyzed at 40-44 hours APF (H-H’’). Pupal wings were stained with DAPI, and in green is shown the activity of TRE-GFP. In control pupal wings the expression of the TRE-GFP reporter is mainly expressed in presumptive hemocytes (yellow arrows in A-A’’’’, C-C’’’’, E-E’’’’ and G-G’’). In irradiated pupal wings, the TRE-GFP reporter is expressed not only in presumptive hemocytes (yellow arrows in B-B’’’’, D-D’’’’, F-F’’’’ and H-H’’) but also in epithelial cells. This observation is supported by the integration of these cells into the epithelium, as evident from DAPI staining (blue arrows in B-B’’’’, D-D’’’’ F-F’’’’ and H-H’’). Images A’’-H’’ correspond to high magnification of the region indicated by a magenta rectangle in images A-H. (I) The graph shows the intensity of the TRE-GFP reporter in non-irradiated and irradiated pupal wings. We only analysed the intensity in the region between vein 4 and 5 to avoid the presence of presumptive haemocytes. Note that the % of cells expressing TRE-GFP is significantly higher in irradiated pupal wings. White scale bar 50μm. Statistical significance was determined using multiple comparation t-Test student (Mann-Whitney test) **** p.

**Additional file 4:** **Fig. S4. **Expression of Wg-GFP in response to damage during pupal development We used the same protocol as described in Fig. 3A to induce the expression of rpr in UAS-rpr; Wg-GFP hh-Gal4; tub-Gal80ts pupae. (A-A’’’, C-C’’’, E-E’’’ and G-G’’’) *Wg-GFP hh-Gal4; tub-Gal80*^*ts*^ control pupal wings examined at developmental stages corresponding to the following time intervals at 25°C: 20- 24h (12h at 17°C+12h at 29°C) (A-A’’’), 25-29h (20h at 29°C) (C-C’’’), 30-34h (12h at 17°C+20h at 29°C) (E-E’’’), and 35-39 h (20h at 17°C+20h at 29°C ) (G-G’’’) APF. (B-B’’’, D-D’’’, F-F’’’ and H-H’’’) UAS-rpr; Wg-GFP hh-Gal4; tub-Gal80^ts^ pupae after 20 h (except for 20-24 h interval see Methods) of rpr overexpression were examined at developmental stages corresponding to the following time intervals at 25°C: 20-24h (12h at 17°C+12h at 29°C) (B-B’’’), 25-29h (20h at 29°C) (D-D’’’), 30-34h (12h at 17°C+20h at 29°C) (F-F’’’), and 35-39 h (20h at 17°C+20h at 29°C ) (H-H’’’) APF. Images A’’-H’’’ correspond to high magnification of the area marked with a magenta rectangle in A-H. The expression of Wg-GFP is shown in green, and DAPI in blue. Note that in damaged pupal wings examined at 20-24 h (yellow arrows in B’’’), and 25-29 h (yellow arrows in D’’-D’’’) Wg-GFP is expressed in regions adjacent to wound healing. In control pupal wings, Wg-GFP is expressed along the wing margin in both the anterior and posterior compartments. White scale bar 50μm.

**Additional file 5:** **Fig. S5.** Expression of *dpp-LacZ* in response to damage during pupal development We used the same protocol as described in Fig. 3A to induce the expression of *rpr* in *UAS-rpr; dpp-LacZ hh-Gal4; tub-Gal80*^*ts*^ pupae. (A-A’’’, C-C’’’, and E-E’’’) *dpp-LacZ hh-Gal4; tub-Gal80*^*ts*^ control pupal wings examined at developmental stages corresponding to the following time intervals at 25°C: 20-24h (12h at 17°C+12h at 29°C) (A-A’’’), 25-29h (20h at 29°C) (C-C’’’), and 30-34h (12h at 17°C+20h at 29°C) (E-E’’’) APF. In control pupal wings *dpp-LacZ* is expressed in a band of cells in the anterior/posterior boundary. (B-B’’’, D-D’’’, and F-F’’’) *UAS*-*rpr; dpp-LacZ hh-Gal4; tub-Gal80*^*ts*^ pupae after 20h (except for 20-24 h interval see Methods) of *rpr* overexpression were examined at developmental stages corresponding to the following time intervals at 25°C: 20-24h (12h at 17°C+12h at 29°C) (B-B’’’), 25-29h (20h at 29°C) (D-D’’’), and 30-34h (12h at 17°C+20h at 29°C) (F-F’’) APF. Images A’’-F’’’ correspond to high magnification of the area marked with a magenta rectangle in A-F. The expression of *dpp-LacZ* revealed with anti- β-Galactosidase is shown in green, and DAPI in blue. (G) Quantification of the width, measured in number of cells, of the band expressing *dpp-LacZ* at the anterior/posterior border in both control and *UAS-rpr; dpp-LacZ hh-Gal4; tubGal80*^*ts*^ pupae wing at 20-24h (12h at 17°C+12h at 29°C). Control *n=*10 and hh>rpr dppZ 20-24h *n=*10. Statistical significance was determined using t-Test student. White scale bar 50μm.

**Additional file 6:** **Fig. S6.** Wg is not essential for the initiation of a proliferative response during pupal development. *UAS-wgRNAi; hh-Gal4 tub-Gal80*^*ts*^ pupae aged 0-4 h were irradiated at 0 hr APF for 8 min and immediately transferred to 29°C for 20 h to induce wgRNAi expression. After this period the pupae were dissected. (A-A’) Control: Non-irradiated *hh-Gal4 tub-Gal80*^*ts*^ pupae kept at 29°C for 20 h. (B-B’) *hh-Gal4 tubGal80*^*ts*^ pupal wing of pupae irradiated at 0h APF and transferred to 29°C for 20 h. (C-C') *UAS-wgRNAi; hh-Gal4 tub-Gal80*^*ts*^*/UAS-GFP* pupal wing kept at 29°C for 20h. (D-D') *UAS-wgRNAi; hh-Gal4 tub-Gal80*^*ts*^ pupal wing irradiated at 0 h APF and kept at 29°C for 20h. Pupal wings were stained with anti-PH3 (red), DAPI (blue). (E) Graph shows the number of mitotic cells (PH3 positive) in the anterior and posterior compartments of pupal wings: hh control: Non-irradiated *hh-Gal4 tub-Gal80*^*ts*^ pupae kept at 29°C for 20 h. IR hh: *hh-Gal4 tub-Gal80*^*ts*^ pupal wing of pupae irradiated at 0h APF and transferred to 29°C for 20h. hh> wgRNAi: *UAS- wgRNAi; hh-Gal4 tub-Gal80*^*ts*^ pupae kept at 29°C for 20 h. IR hh> wgRNAi: irradiated *UAS- wgRNAi; hh-Gal4 tub-Gal80*^*ts*^ pupae irradiated at 0h APF and kept at 29°C for 20h. Statistical analysis was conducted using 2 ways Anova multiple comparation **** p wgRNAi *n=*12 and IR hh> wgRNAi *n=*9. Yellow dotted lines indicate the boundary between the anterior and posterior compartments. White scale bar 50μm.

**Additional file 7:** **Fig. S7.** E2F1 is not a limiting factor for apoptosis- induced proliferation during pupal development To overexpress *rpr* and *E2F1/Dp* in l*exOp-rpr; ci-Gal4; salE/Pv -LHG tub-Gal80*^*ts*^ pupal wings, pupae were collected at 5-hour intervals for staging as described in Methods and then subjected to the following temperature shifts: Developmental stages at 25°C corresponding to 40-45h: (48h at 17ºC + 16 h at 29ºC), and 45-50 h: (60 h at 17ºC + 16 h at 29ºC). (A-A’ and E-E’) Control *ci*-*Gal4; salE/Pv -LHG tub-Gal80*^*ts*^ pupal wings at 40-45 h (48h at 17ºC + 16 h at 29ºC) (A-A’) and 45-50 h (60 h at 17ºC + 16 h at 29ºC) (E-E’). (B-B’ and F-F´) *lexOp*-*rpr; ci-Gal4; salE/Pv -LHG tub-Gal80*^*ts*^ pupal wings at 40-45 h (48h at 17ºC + 16 h at 29ºC) (B-B’) and 45-50 h (60 h at 17ºC + 16 h at 29ºC) (F-F’). (C-C’ and G-G’) *lexOp-rpr; ci-Gal4; salE/Pv-LHG tub-Gal80*^*ts*^ *UAS-E2F1 UAS-Dp* pupal wings at 40-45 h (48h at 17ºC + 16 h at 29ºC) (C-C’) and 45-50 h (60 h at 17ºC + 16 h at 29ºC) (G-G’). (D-D’ and H-H’) *ci-Gal4 tub-Gal80*^*ts*^ *UAS-E2F1 UAS-Dp* pupal wings at 40-45 h (48h at 17ºC + 16 h at 29ºC) (D-D’) and 45-50 h (60 h at 17ºC + 16 h at 29ºC) (H-H’). The discs were stained with anti-PH3 antibody (in green) and Phalloidin to reveal F-Actin (in red). (I) The graphs show number mitotic cells (PH3 positive) in the different experimental condition analyzed. We used the “multiple comparison t-test” to simultaneously analyse the means of the multiple groups used in the assay (Mann-Whitney test) *** *p <* 0.001. 40-45 hours. Anterior compartment: sal>rpr *n=*7, sal>rpr ci>E2F1 Dp *n=*10, ci>E2F1 Dp *n=*9. Posterior compartment sal>rpr *n=*7, sal>rpr ci>E2F1 Dp *n=*10, ci>E2F1 Dp *n=*9. The expression domain of sal is outlined by the white dotted line.

**Additional file 8:** **Fig. S8.** CycE and E2F1 are not a limiting factor for apoptosis- induced proliferation during pupal development (A)Schematic diagram of the irradiation times employed in this experiment. Selected *en-Gal4; tub-Gal80*^*ts*^ *UAS-CycE UAS-GFP* or *en-Gal4; tub-Gal80*^ts^ *UAS-E2F1 UAS-Dp UAS-GFP* pupae aged 0-4 h were irradiated at 0 h APF during 8 min (IR 0 h APF) and transferred immediately to 29°C, or transferred to 29°C and irradiated 20 h later (IR 20 h). The black text indicates the duration of incubation at 29°C, while the red text indicates the equivalent time at 25°C. (B-E) Wings from pupae irradiated at 0 h APF (IR 0 h) and non-irradiated control pupal wings examined 20 h later (20-24 h, corresponding to 25-29 h at 25°C). Non-irradiated *en-Gal4; tub-Gal80*^ts^ *UAS-Cyc E UAS-GFP* (en>Cyc E) (B-B’), *en*-*Gal4; tub-Gal80*^*ts*^ *UAS-Cyc E UAS-GFP* irradiated (IR0h en>Cyc E) (C-C’), non-irradiated *en-Gal4; tub-Gal80*^ts^ *UAS-E2F1 UAS-Dp UAS-GFP* (en>E2F1 Dp) (DD’), and irradiated *en-Gal4; tub-Gal80*ts^ts^ *UAS-E2F1 UAS-Dp UAS-GFP *(IR0h en>E2F1 Dp) (E-E’). (F-I) Wings from pupae incubated during 20 h at 29°C and then irradiated (IR 20 h) and examine 20 h later or control incubated during 40 h but non-irradiated. Both were maintained at 29°C for a total duration of 40 hours (40-44 h, corresponding to 52-56 h at 25°C). Non-irradiated *en-Gal4; tub-Gal80*ts^*ts*^ *UAS-Cyc E UAS-GFP *(en>Cyc E) (F-F’), *en-Gal4; tub-Gal80*^*ts*^ *UAS-Cyc E UAS*-*GFP* irradiated (IR20h en>Cyc E) (G-G’), non-irradiated *en-Gal4; tub-Gal80*^ts^ *UAS-E2F1 UAS-Dp UAS-GFP* (en>E2F1 Dp) (H-H’), and irradiated *en-Gal4; tubGal80*^ts^ *UAS-E2F1 UAS-Dp UAS-GFP* (IR20h en>E2F1 Dp) (I-I’). The wings were stained with anti- PH3 antibody (in red) and Phalloidin to reveal F-Actin (in blue). *UAS-GFP* is shown in green. (J-K) The graphs show the number of mitotic cells (PH3-positive) in both the anterior and posterior compartments of the different genetic variants analysed at 20-24 h (corresponding to 25-29 h at 25°C) for CycE (J) and E2F1 Dp (K). Statistical analysis was conducted using one-way ordinary Anova Tukey’s multiple comparation test **** pCyc E non-irradiated and kept 29°C for 20h (en>Cyc E) *n=*6, en>Cyc E irradiated at 0h and kept 29°C for 20h (en>Cyc E IR 0h) *n=*11. (L) Control 20 h at 29°C (20 h 29C) *n=* 15, Control irradiated at 0h and kept 29°C for 20h (Control IR 0h) *n=*10, en>E2F1 non-irradiated and kept 29°C for 20h (en>E2F1) *n=*7, en>E2F1 irradiated at 0h and kept 29°C for 20h (en>E2F1 IR 0h) *n=*7. The number of wings from pupae incubated during 20 h at 29°C and then irradiated (IR 20 h) and examine 20 h later (40-44 h, corresponding to 52-56 h at 25°C) were: Control 40 h at 29°C *n=* 10, Control irradiated at 20h and kept 29°C for 20h *n=*10, en>CycE non irradiated *n=*7, en>CycE irradiated (IR 20 h) *n=*8, en>E2F1 non irradiated *n=*7, en>E2F1irradiated (IR 20 h) *n=*9. Yellow dotted lines indicate the boundary between the anterior and posterior compartments.

**Additional file 9:** **Fig. S9.** E93 binds temporally dynamic open chromatin. (A) Browser shot from the stg, CycE and E2F1 loci showing FAIRE-seq and E93 ChIP-seq (in blue) signals (Z-score) score representation from wildtype (Wt L3, WtL 24 h APF, WtL 44 APF) and E93 mutant (E93L3, E93 24 APF, E93 44 APF) samples. Peaks are shown in green and red, respectively. The analysis spans larval stages (Wt L3 and E93L3), 24 h APF pupal wings (WtL 24 h APF and E93 24 APF), and 44h old pupal wings (WtL 44 APF and E93 44 APF). Notably, certain peaks representing open chromatin exhibit decreased or vanished signals in wildtype pupae at 44 h, while in E93 mutants, these regions persist or only show slight reduction (indicated by yellow bands). Data obtained from [18].

## Data Availability

The data and materials supporting the findings of this study are available upon request. Interested parties may contact the corresponding author to obtain access to the data and materials for further examination and verification.

## Abbreviations

APF: After puparium formation
DBS: Drice Base sensor
dpp: Decapentaplegic
h: Hours
JNK: C-Jun N-terminal kinases
ROI: Region of interest
*Stg*: *string*
Wg: Wingless
