# Supplementary figures and images for: Temporal dynamics of apoptosis-induced proliferation in pupal wing development: implications for regenerative ability
Source: BMC Biol. 2024 Apr 29;22:98. doi: 10.1186/s12915-024-01894-1 (PMC11057159; doi:10.1186/s12915-024-01894-1)

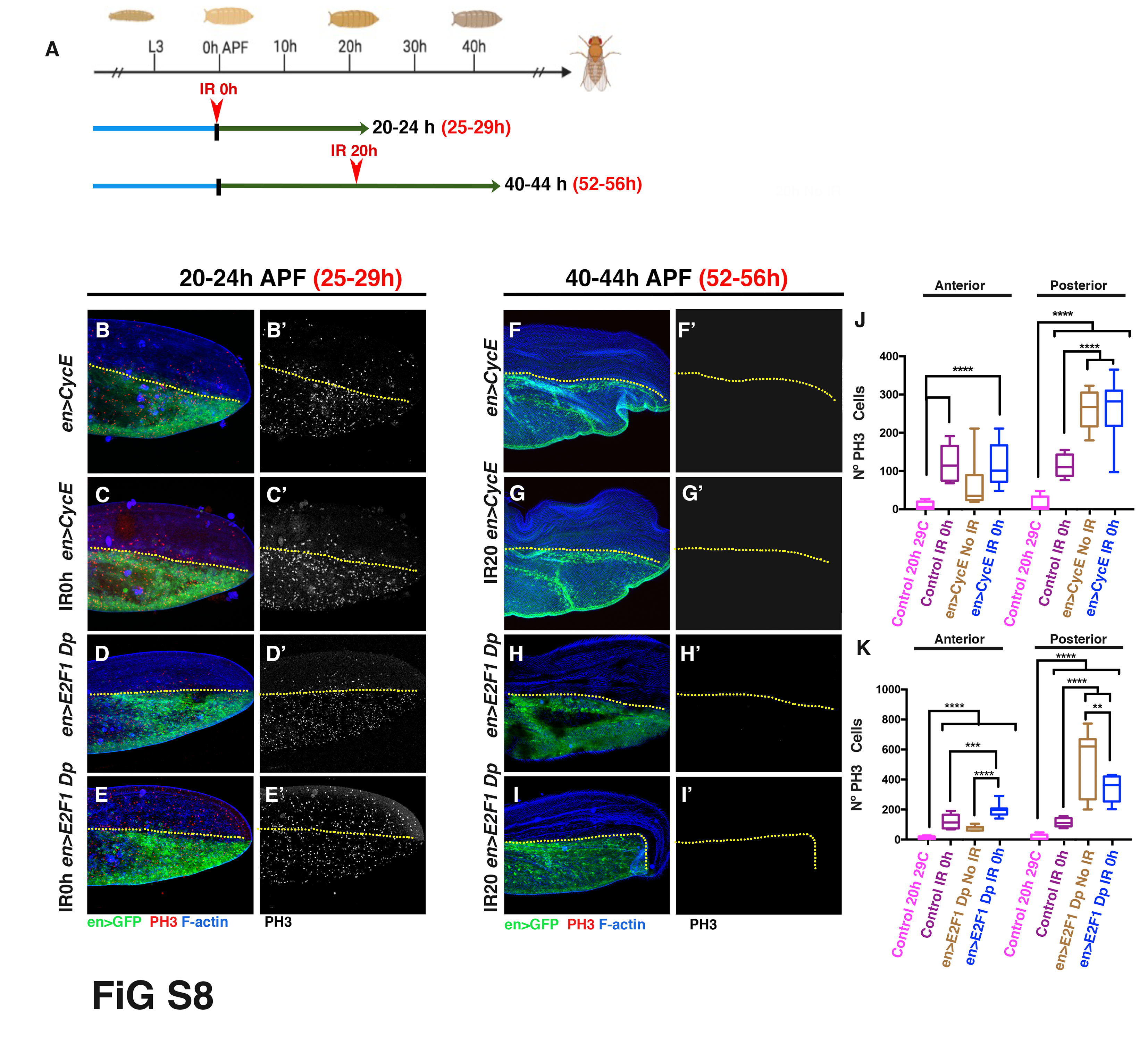

Supplement: Supplementary file 8 — Additional file 8: Fig. S8. CycE and E2F1 are not a limiting factor for apoptosis- induced proliferation during pupal development (A)Schematic diagram of the irradiation times employed in this experiment. Selected en-Gal4; tub-Gal80ts UAS-CycE UAS-GFP or en-Gal4; tub-Gal80ts UAS-E2F1 UAS-Dp UAS-GFP pupae aged 0-4 h were irradiated at 0 h APF during 8 min (IR 0 h APF) and transferred immediately to 29°C, or transferred to 29°C and irradiated 20 h later (IR 20 h). The black text indicates the duration of incubation at 29°C, while the red text indicates the equivalent time at 25°C. (B-E) Wings from pupae irradiated at 0 h APF (IR 0 h) and non-irradiated control pupal wings examined 20 h later (20-24 h, corresponding to 25-29 h at 25°C). Non-irradiated en-Gal4; tub-Gal80ts UAS-Cyc E UAS-GFP (en>Cyc E) (B-B’), en-Gal4; tub-Gal80ts UAS-Cyc E UAS-GFP irradiated (IR0h en>Cyc E) (C-C’), non-irradiated en-Gal4; tub-Gal80ts UAS-E2F1 UAS-Dp UAS-GFP (en>E2F1 Dp) (DD’), and irradiated en-Gal4; tub-Gal80tsts UAS-E2F1 UAS-Dp UAS-GFP (IR0h en>E2F1 Dp) (E-E’). (F-I) Wings from pupae incubated during 20 h at 29°C and then irradiated (IR 20 h) and examine 20 h later or control incubated during 40 h but non-irradiated. Both were maintained at 29°C for a total duration of 40 hours (40-44 h, corresponding to 52-56 h at 25°C). Non-irradiated en-Gal4; tub-Gal80tsts UAS-Cyc E UAS-GFP (en>Cyc E) (F-F’), en-Gal4; tub-Gal80ts UAS-Cyc E UAS-GFP irradiated (IR20h en>Cyc E) (G-G’), non-irradiated en-Gal4; tub-Gal80ts UAS-E2F1 UAS-Dp UAS-GFP (en>E2F1 Dp) (H-H’), and irradiated en-Gal4; tubGal80ts UAS-E2F1 UAS-Dp UAS-GFP (IR20h en>E2F1 Dp) (I-I’). The wings were stained with anti- PH3 antibody (in red) and Phalloidin to reveal F-Actin (in blue). UAS-GFP is shown in green. (J-K) The graphs show the number of mitotic cells (PH3-positive) in both the anterior and posterior compartments of the different genetic variants analysed at 20-24 h (corresponding to 25-29 h at 25°C) for CycE (J) and E2F1 Dp (K). [file 12915_2024_1894_MOESM8_ESM.tif]

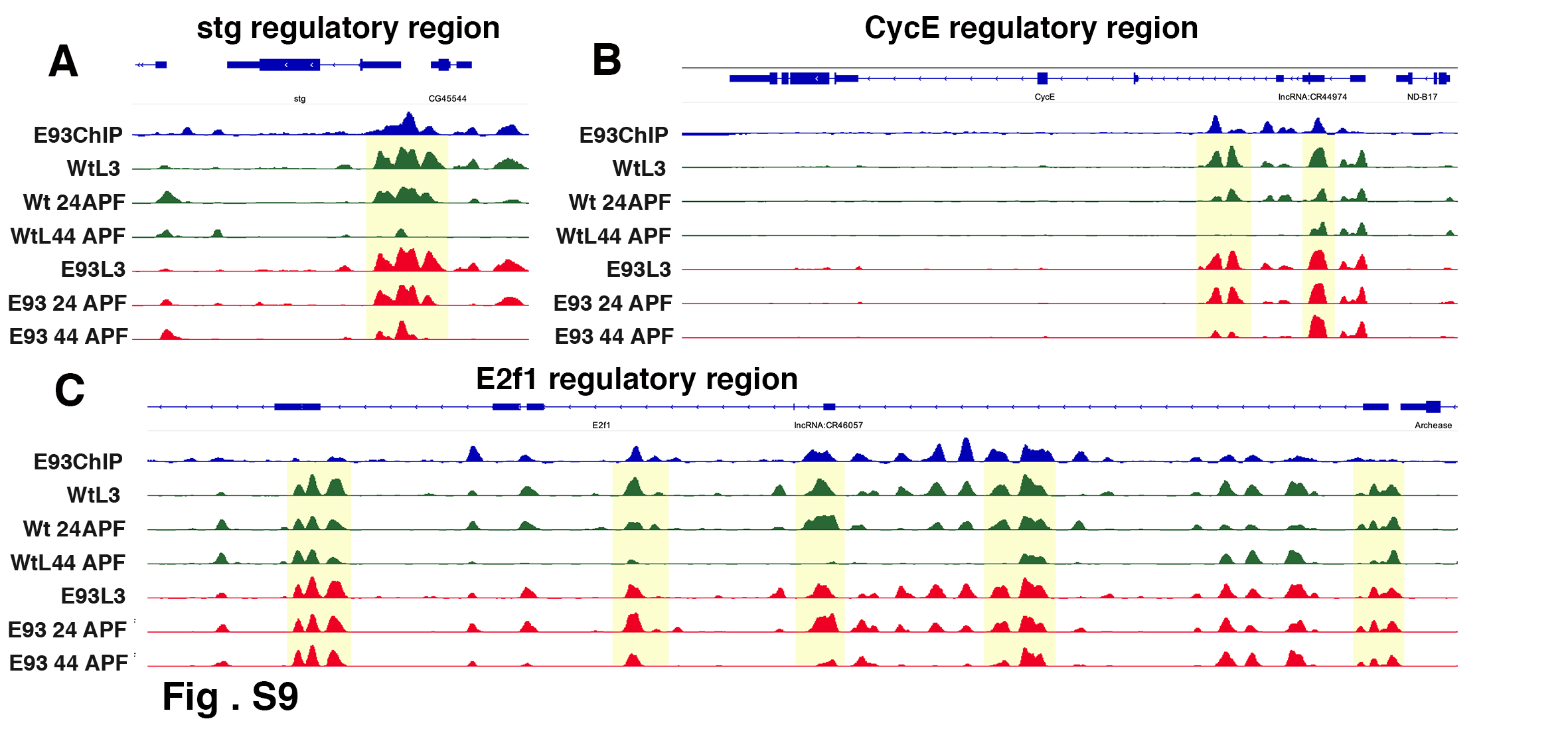

Supplement: Supplementary file 9 — Additional file 9: Fig. S9. E93 binds temporally dynamic open chromatin. (A) Browser shot from the stg, CycE and E2F1 loci showing FAIRE-seq and E93 ChIP-seq (in blue) signals (Z-score) score representation from wildtype (Wt L3, WtL 24 h APF, WtL 44 APF) and E93 mutant (E93L3, E93 24 APF, E93 44 APF) samples. Peaks are shown in green and red, respectively. The analysis spans larval stages (Wt L3 and E93L3), 24 h APF pupal wings (WtL 24 h APF and E93 24 APF), and 44h old pupal wings (WtL 44 APF and E93 44 APF). Notably, certain peaks representing open chromatin exhibit decreased or vanished signals in wildtype pupae at 44 h, while in E93 mutants, these regions persist or only show slight reduction (indicated by yellow bands). Data obtained from [18]. [file 12915_2024_1894_MOESM9_ESM.tif]
